# Adsorption of serum components on Ag colloids: on the biochemical interpretation of surface-enhanced Raman spectra of human serum

**DOI:** 10.1007/s00216-025-06192-5

**Published:** 2025-11-17

**Authors:** Roberto Gobbato, Stefano Fornasaro, Valter Sergo, Alois Bonifacio

**Affiliations:** 1 Raman Spectroscopy Laboratory, Department of Engineering and Architecture, University of Trieste, Via Valerio 6a, 34127 Trieste, TS Italy; 2 Department of Chemical and Pharmaceutical Sciences, University of Trieste, Via Licio Giorgieri 1, 34127 Trieste, TS Italy

**Keywords:** SERS, Surface-enhanced Raman, Uric acid, Hypoxanthine, Biofluids, Human serum albumin

## Abstract

Surface-enhanced Raman scattering (SERS) spectra of human serum exhibit consistent features across studies from multiple research groups, while the biochemical interpretation of bands has not yet reached a consensus. By critically reviewing the recent literature, the methods commonly used to assign SERS bands in biofluids are re-evaluated. Data are presented from various experimental approaches to test band attribution. Analyses of serum samples spiked with various biomolecules, spectral changes induced by uricase treatment, correlation patterns among bands across a serum dataset, and spectral fitting all indicate that the primary contributors to the SERS spectrum of serum (at its unaltered physiological pH of 7.4), obtained on Ag substrates with near infrared excitation, are uric acid and hypoxanthine. Principal component analysis further reveals that most of the dataset variance—reflecting inter-individual biochemical differences—is associated with the bands of these two metabolites. Additionally, data from isotopically labeled uric acid demonstrate that both its albumin-bound and free forms, which exhibit distinct spectral signatures, contribute to the overall serum spectrum.

**Graphical Abstract:**

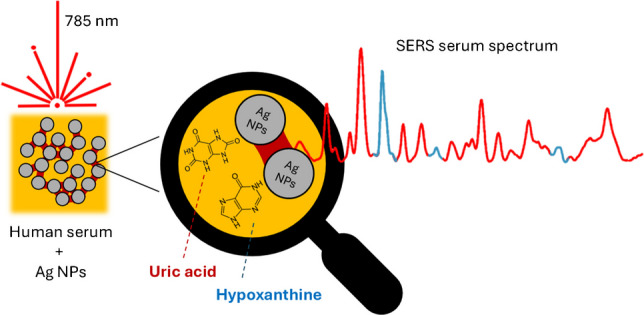

**Supplementary Information:**

The online version contains supplementary material available at 10.1007/s00216-025-06192-5.

## Introduction

The application of surface-enhanced Raman spectroscopy to biofluids, particularly to serum and plasma, using a direct and untargeted analytical approach has been the object of intense research for more than a decade. The main scope of this activity has been the diagnosis and/or staging of specific pathologies. Despite differences in substrates, protocols, and instruments used, SERS spectra of serum obtained by different research groups share the same spectral pattern when using a near infrared excitation (785 nm) with Ag substrates (this being the most common experimental configuration) [1]. Absolute and relative intensities might vary across different studies, but the overall structure of spectra is conserved, with most of the bands being reported always at the same Raman shifts, within minor variations. This ubiquitous spectral pattern supports the idea that a SERS approach to the direct analysis of serum is repeatable.

On the other hand, despite this convergence of experimental data, their biochemical interpretation, i.e., the attribution of each band to a specific biomolecule, has not reached a general consensus yet. In other words, different authors report the same bands while giving them very diverse interpretations. This lack of consensus is critical, since any discussion about the biochemistry at the basis of the reported spectral differences is unreliable and generates confusion. In some cases, different authors report consistent spectral differences between two sample groups while giving them a very different biochemical interpretation. For instance, while studying hepatocellular carcinoma, two research groups [2, 3] independently reported that SERS spectra of serum from patients with cancer had a higher intensity ratio between bands at 638 and 724 cm^−1^ with respect to the control group, but the interpretation of this feature was very different. One group interpreted the data as indicating that the cancer patients had relatively higher serum levels of tyrosine and lactose with concurring lower levels of coenzyme A when compared to the healthy controls. The other group interpreted the same spectral trend attributing to the cancer patients a higher uric acid/hypoxanthine ratio relative to the healthy controls. This example is paradigmatic of the general disagreement about the biomolecular interpretation of SERS spectra of serum and how it can have possible clinical consequences when looking for biomarkers. The persistence, as of today, of such lack of consensus is a serious problem for the credibility of untargeted SERS of serum as a diagnostic tool, as it hinders any reliable biochemical interpretation of the ever-growing amount of spectroscopic data across many different medical conditions. A persistent incorrect interpretation, when further propagated, might mislead clinical researchers studying a particular disease, suggesting incorrect metabolic differences among groups of patients.

For this reason, we believe that it is important to settle the issue about the biochemical meaning of the bands recurring in direct SERS studies on serum. The goal of the present study is indeed to unambiguously identify the biomolecules associated with the SERS bands of serum by providing direct experimental evidence obtained using different approaches.

## Materials and methods

### Literature analysis

A literature search was performed on the SCOPUS and PubMed databases using as query (“SERS” OR “Surface enhanced Raman”) AND “serum” AND “silver” for papers published from 2015 to 2023. A further sub-selection was done according to the following inclusion criteria: (i) the paper must report at least one serum SERS spectrum of a healthy individual, obtained with a label-free (i.e., direct) detection strategy and using a NIR laser (at 785 nm); (ii) the spectra reported must be clearly presented, without artifacts due to data post-processing (e.g., baseline subtraction); (iii) SERS effect must be provided by Ag nanoparticles, AgNP, in colloidal form or deposited onto solid substrates.

Information for each article in the dataset is summarized in a worksheet (see Supplementary Material files – Table S1), where the value of each band position (Raman shift, cm^−1^) has been reported alongside with the correspondent interpretation. Citation networks (see Supplementary Material 1 - Figures S1, S2) were then manually extracted for two representative serum bands, namely those in the 630–640 cm^−1^, and 721–730 cm^−1^ ranges, and saved in the form of a numerical directed matrix used for plotting the network graphs (Supplementary Material 2, Supplementary Material 3). The citation networks obtained contain three types of nodes: (i) starting nodes (most recent papers); (ii) intermediate nodes (the ones linked to the starting nodes, that are still citing other papers); and (iii) end nodes. The latter are defined as the papers in which no other citation was provided in support of the assignment (i.e., dead ends), or in which Raman or SERS experimental data (i.e. a spectrum of the pure putative biomolecule) was reported.

### Reagents and solvents

Analytical grade silver nitrate (AgNO_3_; CAS No. 7761-88-8), sodium citrate tribasic dihydrate (Na_3_C_6_H_8_O_7_·2H_2_O; CAS No. 6132-04-3), used for the nanoparticle synthesis were purchased from Merck. Ultrapure water (MilliQ), sodium hydroxide (NaOH; CAS No. 1310-73-2), hydrochloric acid (HCl; CAS No. 7647-01−0), phosphate-buffered saline (PBS) and ethanol (CH_3_CH_2_OH; CAS No. 64-17−5) were employed to prepare stock solutions, as well as solutions at average physiological concentrations, of the biomolecules used in this study (see Supplementary Material 1- Figure S3 for preparation procedures).

The complete list of the biomolecules used is presented in Supplementary Material – Table S2.

Uricase of *Bacillus fastidiosus* (CAS No. 9002-12−4) was purchased from Merck.

Isotopically substituted uric acid (uric acid-2-^13^C,1,3,7-^15^N_3_) was purchased from Merck, and commercially available Vivaspin® ultrafiltration spin columns with a molecular weight cutoff of 3 kDa (Sartorius) were employed for the deproteinization steps.

### Commercial serum and serum samples from donors

Commercial human serum (from male AB clotted whole blood, USA origin, sterile filtered) was purchased from Merck (product number H6914), aliquoted in 1 mL Eppendorfs and frozen at −30°C to prevent degradation over time. Blood serum aliquots (200 μL) were collected from the blood donated to Blood Donation Center (Transfusion Medicine Unit) at the “Maggiore” hospital in Trieste by 81 healthy volunteers. All samples were anonymized. Informed consent was obtained from all donors to ensure they understood that part of their blood would have been used for research purposes, before blood donation. All serum samples were prepared according to usual blood collection protocols (EDTA blood collection tubes, centrifugation) and stored at −30°C until analysis.

### Citrate-reduced Ag colloid preparation

Citrate-reduced AgNP were synthesized according to the Lee-Meisel method [4]. Briefly, 90 mg of silver nitrate were dissolved in 500 mL of milliQ water and heated until boiling. 10 mL of a 1.1% w/v sodium citrate tribasic solution were added dropwise under continuous magnetic stirring. The solution was kept boiling under stirring for 1 h, then left cooling to room temperature and stored in the dark for later use. 100 μL of the colloidal solution were diluted 10 times in MilliQ water, placed in a PMMA cuvette and characterized by UV-Visible absorption spectroscopy using a Lambda 20bio UV-Vis spectrometer (Perkin-Elmer Italia, Monza, Italy). In agreement with values previously reported in literature [5], AgNP featured a surface plasmon band at 410 ±5 nm.

### Protocols for spiking commercial serum

Commercial serum sample was taken out of the freezer at −30°C and thawed at room temperature, and then, 49 μL was transferred in a new Eppendorf tube. For each biomolecule cited in the list of articles considered (see Supplementary Material – Table S1) a concentrated 10 mM solution was prepared using the proper solvent to dissolve completely the biomolecular species; the solution was then diluted to 1 mM concentration using PBS, from which a solution with 50 times the average physiological concentration was prepared by further dilution in PBS. The concentration of the latter solution was chosen to ensure that the spike of 1 μL in 49 μL of serum corresponded to a final concentration in accordance with the average physiological values reported in the literature [6]. For biomolecules with relatively high physiological concentrations (e.g., uric acid, glucose, glycine, urea, alanine, cholesterol), it was necessary to prepare a starting spiking solution with a concentration higher than 10 mM. Additional details of the procedure are summarized in Supplementary Material 1- Figure S3.

### Isotopically substituted uric acid experiment

A 10 mM stock solution of isotopic uric acid was prepared dissolving 1 mg in 581 μL of NaOH 1 M; the stock solution was then diluted to 1 mM and then to the respective physiological concentration (280 μM) both using a PBS solution, and a 50 mg/mL solution of human serum albumin (HSA) dissolved in PBS. 200 μL of the 280 μM uric acid in HSA were then ultracentrifuged with a Vivaspin® ultrafiltration spin column at 19,600 g for 20 min. After the centrifugation, the remaining solution that did not pass through the filter was taken and resuspended in PBS to gain the same initial volume of the starting solution (200 μL).

The same steps were repeated also with non-labeled uric acid to gain reference spectra. The following solutions were analyzed with the SERS measurement protocol described later : 280 μM uric acid (normal and labeled) in PBS, 280 μM uric acid (normal and labeled) in HSA 50 mg/mL, filtered 280 μM uric acid (normal and labeled) in HSA, unfiltered and resuspended solution (normal and labeled).

### Uricase experiments

5 mg of lyophilized uricase were resuspended in 1 mL PBS solution to obtain an enzymatic stock solution of 45 U/mL. A 10-fold dilution was performed taking 100 μL of the stock solution and transferring it in 900 μL of PBS. 1 μL of this diluted solution was added directly to 50 μL of commercial serum, and to 50 μL of commercial serum spiked with hypoxanthine to deplete the samples of uric acid. The samples were then left at room temperature for 15 min and analyzed with the protocol presented in the following section. For more details about the procedure, see Supplementary Material 1 - Figure S4.

### SERS and Raman measurements

The protocol for SERS analysis used in this work consisted in directly mixing a serum sample with concentrated AgNP, and then drying the mixture on a CaF_2_ slide (see Supplementary Material 1 - Figure S5). More in detail, 1 mL of Ag was centrifuged for 10 min at a relative centrifugal force of 25,500 g. The Ag colloids were then concentrated discarding 900 μL of the supernatant and resuspending the nanoparticle pellet in the remaining 100 μL. 5 μL of the concentrated solution was mixed with 5 μL of the sample of interest, and then 5 μL of the mixture were deposited on the CaF_2_ slide and analyzed in the “coffee-ring” region after 20 min of drying at room temperature with a laser power of 10 mW.

Commercial serum samples (non-spiked and spiked with different biomolecules), donors serum samples, the sample treated with uricase and the pure biomolecules solutions at different concentrations (1 mM and physiological concentration) were all analyzed with the aforementioned protocol, because (i) it is the most widely used in the literature, (ii) it does not need any deproteinization step for the preparation of the serum samples (avoiding the costs of the ultracentrifugation filters), and (iii) it has a low spectral variability when compared with other protocols [1]. Additionally, to evaluate the differences between SERS spectra of pure substances with the corresponding normal Raman spectrum, powder of all analytes were analyzed by depositing them on a glass microscope slide covered with aluminum foil. For all samples the analysis was performed with a B&WTek i-Raman Plus portable system (BWS465-785S) connected to a compatible Raman microscope (BAC151B) mounting a ×20 Olympus objective (N.A. 0.4). Sample illumination and scattering light collection was obtained using optical fibers connected to the Raman microscope with a CleanLaze® stabilized laser system model with a wavelength of 785 nm and a maximum laser power of 400 mW.

### Data preprocessing and analysis

All data preprocessing, data analysis and figure preparation were made with the R software [7], using the *hyperSpec* package [8] to manage spectral data. A custom import function *scan.txt.BWtek* was used to import raw data (.txt ASCII files) into a single *hyperSpec* S4 object. Metadata were extracted from the filename and attached to the hyperspec object. Spectral region was cropped for all spectra to the 400–1800 cm^−1^ range. Baselines were calculated and subtracted for all spectra with the “*modpolyfit*” algorithm of the *baseline* function (package *baseline* [9]), using the degree = 4. Intensity normalization was obtained by dividing each spectrum by a factor calculated as sqrt(sum(x_i_^2^)), where x_i_ were the intensity values at each data point (vector normalization).

Band maxima were automatically detected and labeled using the *detectPeaks* function from the *MALDIquant* package [10], using the “*MAD*” and “*SuperSmoother*” methods.

Spearman correlation coefficients among different bands were calculated using the *cor* function of base R.

Spectral decomposition was performed by fitting a target spectrum as a linear combination of known component spectra (i.e., uric acid, both free and HSA-bound, and hypoxanthine), allowing for both amplitude scaling and small horizontal (x-axis) shifts of each component. Fitting was conducted using the *optim* function of the *stats* package with the “*L-BFGS-B*” method [11], which permits explicit bounds on parameters. Specifically, amplitude coefficients were constrained to non-negative values, and shifts were restricted to ±0.1 cm^−1^. The fitting minimized the sum of squared residuals between the observed and reconstructed spectra. Component interpolation following shift was performed using linear interpolation (*approx* function). The 95% confidence intervals for the figures of merit to assess the goodness of fit (i.e., R^2^, normalized Root Mean Square Error, RMSE and reduced χ^2^) were calculated using a non-parametric bootstrap approach (1000 bootstrap resamples) with the *boot.ci* function of the *boot* package [12].

PCA was done using the *prcomp* function, centering but not scaling data.

Figures were prepared using base R plotting functions.

## Results and discussion

### Literature analysis of bands interpretation

**Fig. 1:**
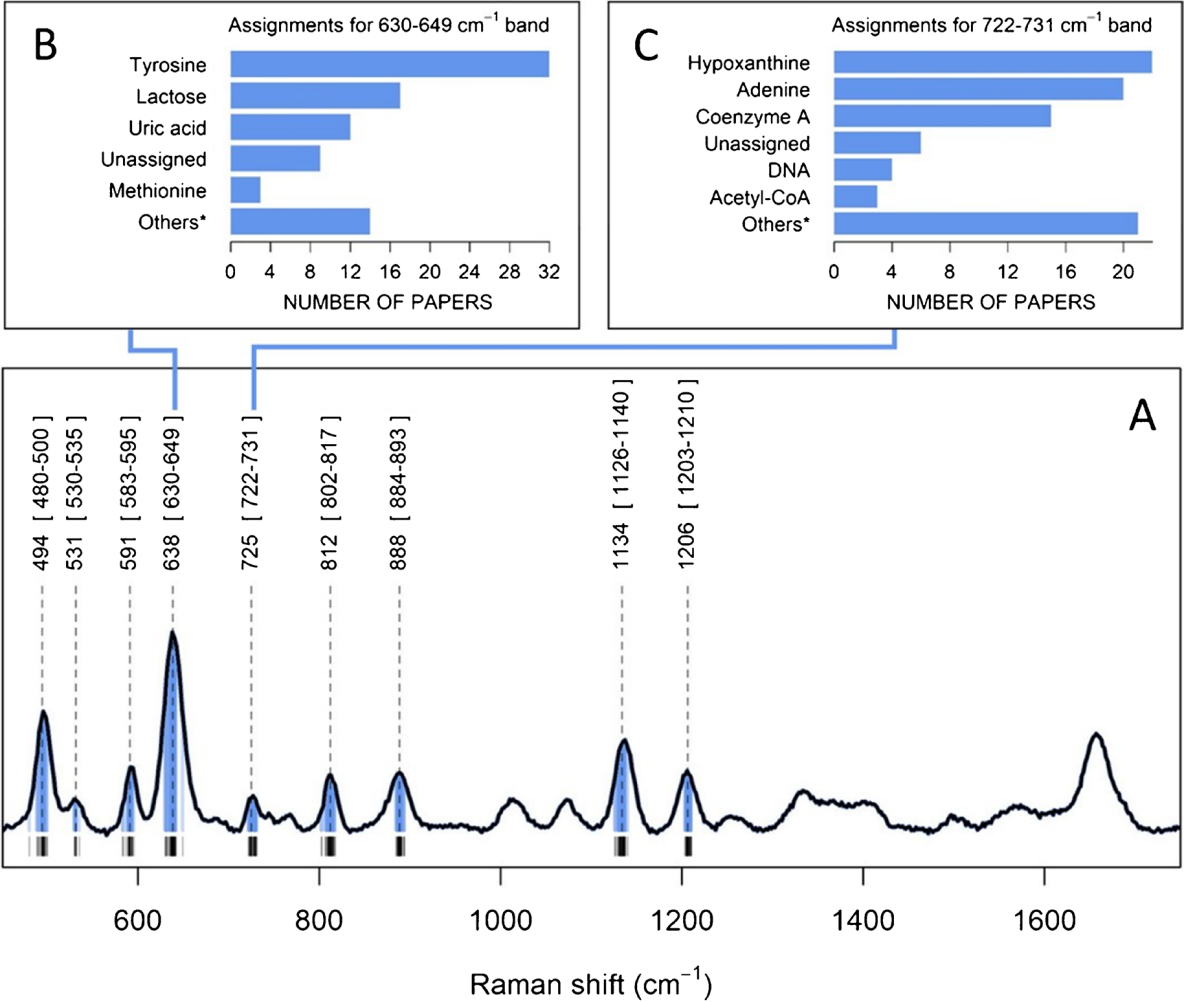
**A** SERS spectrum of commercial serum, representative of the spectra reported in literature. Average band maxima (calculated from literature data), together with minimum and maximum values, are shown for most bands commonly reported. Black lines at the bottom and blue lines under the spectrum show the positions of each band for all the papers considered in the analysis; **B**, **C** bar plots reporting the number of papers assigning a specific biomolecule to bands at 638 and 725 cm.^−1^, respectively. Only the most frequently assigned biomolecules are shown; all those biomolecules occurring in less than 3 papers are grouped as “others” (complete bar graphs for all the bands are shown in Supplementary Material 1 – Figure S6)

Figure 1A reports an example of a SERS spectrum of serum with the characteristic pattern of bands reported in literature. Bands that are most frequently the object of a biochemical interpretation are labeled with a Raman shift value that is the median value of those reported in the papers included in our analysis (see “Materials and methods” for details), together with the minimum and maximum values. Bar plots detailing the various biochemical attributions are shown for the bands at 638 cm^−1^ and at 725 cm^−1^ (Figure 1B, C). These two bands are often found to consistently play an important role in spectral differences among different groups (e.g., disease vs control). Complete bar plots for all the bands are reported in Supplementary Material 1- Figure S6. This analysis highlights the discrepancies among band interpretations found in literature. For instance, the band observed at 638 cm^−1^ (usually the most intense band reported) has been mostly attributed to tyrosine, but also to lactose, uric acid and, in few papers, to methionine.

To better understand the reasons behind this situation, it is worth considering the evidence that the various authors provided to support their interpretation. In principle, the attribution of a SERS band to a specific biomolecule, in the spectrum of a complex mixture such as serum, should be done based on first-hand evidence. That is, a direct comparison between the spectra of serum and that of the biomolecule, obtained with the same experimental conditions (i.e., same substrate, same excitation laser, same data collection protocol). To take into account matrix effects on SERS signal (e.g., ionic strength, pH, interference of other components, etc.), a more robust strategy would be to spike serum with a small volume of a solution of the biomolecule of interest, looking for increase in relative intensity of the SERS spectrum obtained from the spiked sample. Citing a paper that reports a SERS spectrum of the biomolecule of interest obtained in similar experimental conditions is also acceptable, if the spectral pattern found in literature is approximately corresponding to some of the bands observed in serum. Limiting the comparison to just one band, based on a similar Raman shift, and neglecting the occurrence of other bands, is clearly misleading, as it arbitrarily isolates one part of the spectrum from the rest. This habit falls within the so-called fallacy of incomplete evidence, or “cherry picking”, where the analysis is focused on specific data that seems to confirm a hypothesis, ignoring the rest of the data that may contradict it.

With the exception of very few cases, the method most frequently used to support the biochemical interpretation of a band is to refer to other papers, which in principle might be a reasonable strategy, if the papers cited showed convincing evidence for that interpretation (see the cases listed above). However, an analysis of the citation network (see Supplementary Material 1 - Figure S1, S2) reveals that in most cases the cited papers do not contain experimental evidence. Instead, they further refer to other papers, which refer to previous papers, and so on, reiteratively delegating the burden of proof to others. Ultimately, “citation chains” are created, having the effect of obscuring the experimental evidence.

In many cases, these citation chains eventually lead to papers reporting not SERS but normal Raman spectra of the biomolecule of interest. The use of a normal Raman spectrum as the reference to assign a SERS band is problematic, as the Raman and SERS spectra of the same analyte can be remarkably different both in terms of band position and relative intensity (see Supplementary Material 1 for a comparison between SERS – Figure S7 - and Raman – Figure S8 - spectra). This difference is a direct consequence of the metal-molecule interaction occurring in SERS [13]. Regardless of whether the paper at the end of the citation chain reports a SERS or a normal Raman spectrum, a common issue is that the comparison is limited to a single band having a similar position, disregarding the overall spectral pattern (see the “cherry picking” fallacy previously described).

**Fig. 2:**
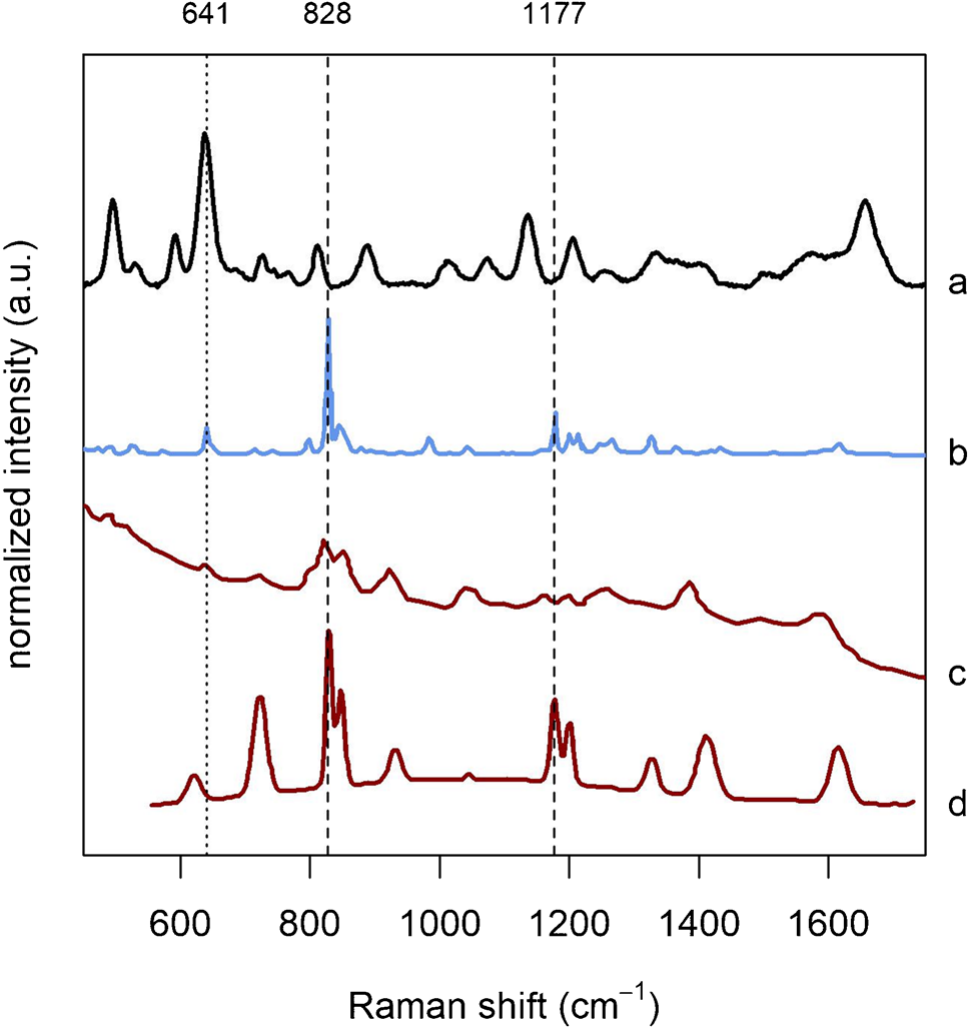
**a** SERS spectrum of serum on AgNP @785 nm, **b** normal Raman spectrum (@785 nm) of tyrosine from [18], **c** SERS spectrum of tyrosine on AgNP @514.5 nm from [19], **d** SERS spectrum of tyrosine on Ag @632.8 nm from [20]. Spectra **b**, **c**, and **d** were obtained by using Plot Digitizer (https://plotdigitizer.com/)

The methodological issues affecting SERS band interpretation are exemplified by the attribution of the 638 cm^−1^ serum band to tyrosine (i.e., the most common interpretation for that band, see Fig. 1B). Many authors (e.g., [14–17]) adopt the citation chain practice, eventually leading to the study by de Gelder *et al.* (see the citation network graph of Supplementary Material 1- Figure S1), a highly cited paper that reports normal Raman spectra of various biomolecules, including tyrosine [18]. In this paper, the normal Raman spectrum of tyrosine has indeed a band at 641 cm^−1^, close to that at 638 cm^−1^ of the SERS spectrum of serum (Fig. 2a, b, dotted line). Interestingly, also the Raman spectra of citric acid, proline, pyruvate and glutathione reported in that paper all show a band close to 638 cm^−1^, but for some unexplained reason they were not considered as candidates. The Raman spectrum of tyrosine reported by de Gelder *et al.*, however, also has a very intense band at 828 cm^−1^ (five times more intense than the one at 641 cm^−1^), and a medium-intensity band at 1177 cm^−1^ (see dashed lines in Fig. 2). If tyrosine is supposed to contribute to the serum SERS spectra, based on its normal Raman spectrum, then bands close to 828 cm^−1^ and 1177 cm^−1^ should appear there as well. However, no bands in those regions are present in the SERS spectra of serum reported in the studies considered in this analysis, suggesting that the normal Raman spectrum of tyrosine cannot be taken as solid evidence to support such an attribution.

To support their assignment of the 638 cm^−1^ band to tyrosine, other authors (e.g., [21, 22]) refer to studies directly or indirectly reporting SERS spectra of tyrosine [19, 20], obtained using excitation wavelengths other than 785 nm. Even in those cases the situation is the same as before: those SERS spectra have weak bands close to 638 cm^−1^ (Fig. 2c, d), but their overall spectral profiles are incompatible with that of serum, suggesting that they cannot be taken as an evidence to support an assignment of the 638 cm^−1^ band to tyrosine.

The case reported for tyrosine is representative for many other putative biomolecules attributed to the 638 cm^−1^ SERS band of serum, and the same situation is replicated for the other bands as well (as an example, see the citation graph for the 725 cm^−1^ band in Supplementary Material 1- Figure S2). The habit of using citation chains (often leading to normal Raman spectra) and the practice of neglecting the overall spectral pattern by focusing on a single band are critical methodological issues recurring in the vast majority of the papers considered in this study. We believe that such flawed methods for band assignment concurred to the present situation, leading to conflicting interpretations for SERS bands or serum.

### SERS spectra of individual biomolecules

**Fig. 3:**
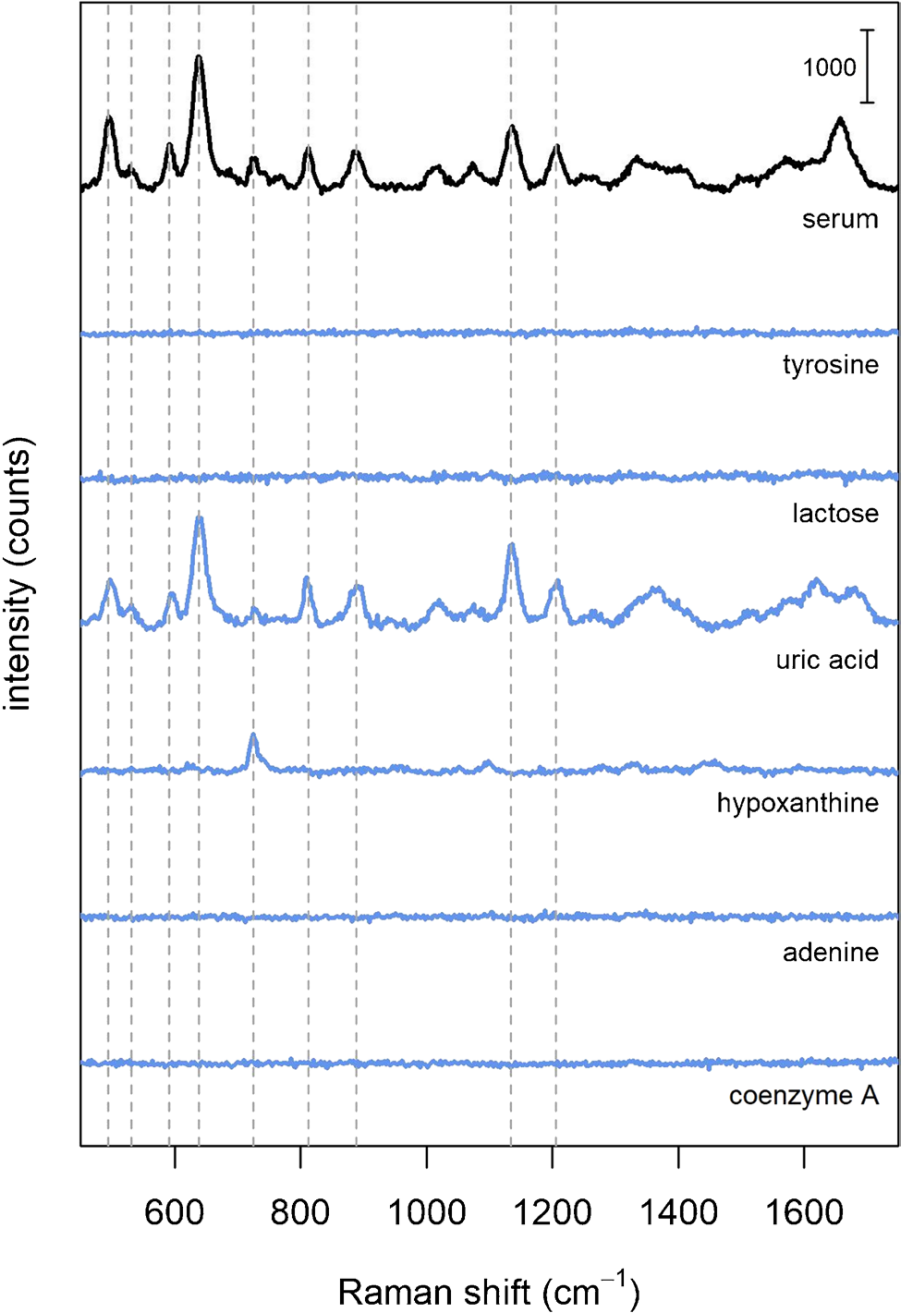
SERS spectrum of commercial serum compared to SERS spectra of selected biomolecules: tyrosine (55 μM), lactose (100 μM), uric acid (280 μM), hypoxanthine (2.3 μM), adenine (0.3 μM) and coenzyme A (100 μM). Biomolecules’ concentrations were chosen to match physiological ranges, as from [6]. The Raman shifts of main serum bands (as shown in Fig. 1) are marked with dashed gray lines

As already stated, a simple method to validate a hypothesis on a SERS band interpretation for complex mixtures is to directly compare the spectrum of the mixture with that of the alleged component, where both spectra have been obtained by using exactly the same protocol for SERS analysis. Figure 3 shows the spectrum of commercial serum together with those of some biomolecules (from PBS solutions at physiological ranges), all obtained by using the same protocol. The protocol adopted is the one most frequently used in the papers included in our literature analysis: whole serum is mixed with AgNPs, and then the spectrum is acquired upon drying and formation of the “coffee-ring” structure [1] (see “Materials and methods” for details). The spectra shown as examples in Figure 3 are from those biomolecules which were most frequently attributed to the bands at 638 and 725 cm^−1^ (see Figure 1B, C). SERS and normal Raman spectra for other biomolecules are reported in the Supplementary Material 1 - Figure S7, S8.

The data show how the only metabolite that yields an intense SERS spectrum is uric acid, while hypoxanthine shows a weak band at 725 cm^−1^. Tyrosine and lactose do not yield any SERS signal, and therefore they are unlikely to contribute to the 638 cm^−1^ serum band, nor to any other band. On the other hand, the same data suggest that uric acid is the metabolite responsible for the 638 cm^−1^ band, and for most of the bands in the SERS spectrum of serum. This interpretation has been first proposed by our research group in 2014 [23], based on a direct spectral comparison between serum and uric acid. Despite this spectral similarity, however, most authors did not follow this interpretation, and continued to assign serum bands to other biomolecules, not based on direct experimental evidence but referring to other studies. The fact that the SERS spectrum of a complex and rich mixture such as serum (with more than 4000 metabolites [24]) is mostly due to just one metabolite might be comprehensibly difficult to accept. It might appear as counterintuitive to many analytical scientists, but this fact can be rationalized taking into account specific aspects of SERS as a surface technique. The data presented in this work, showing how intense SERS spectra are obtained from just few biomolecules out of the 53 tested (see Supplementary Material 1), confirms this assumption. Differently from other spectroscopic techniques used for chemical analysis, the intensity of the SERS signal of an analyte is a direct consequence of its interaction with the metal surface of the substrate. In a mixture, the affinity of an analyte for the metal is far more important than its concentration in determining which components will appear in the SERS spectrum. Glucose, for instance, despite being one of the blood metabolites with the highest concentration (i.e., in the millimolar range), is very difficult to be directly detected with SERS [25], not only because of its low Raman scattering cross section, but mainly because of its low affinity for Ag and Au substrates. Notwithstanding the initial hesitancy, however, this interpretation of serum SERS bands as due to uric acid has been corroborated by other research groups, upon directly comparing uric acid and serum spectra [26, 27].

### Free and protein-bound uric acid have different spectra

**Fig. 4:**
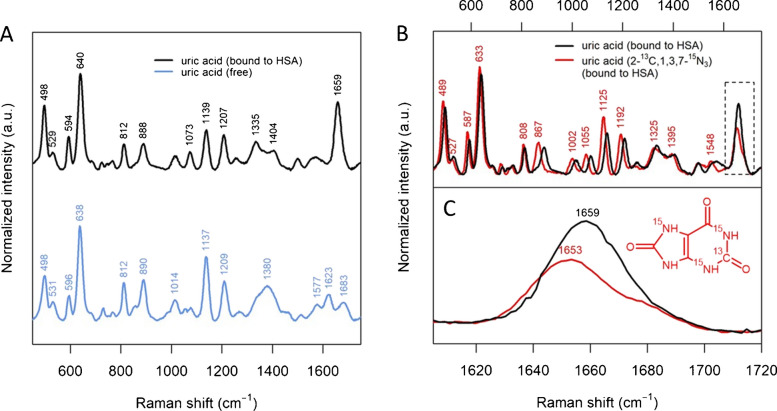
(**A**) SERS spectra of uric acid (280 μM in PBS), free (bottom, blue line) and bound to HSA (top, black line); (**B**) SERS spectra of unmodified (black line) and isotopically substituted uric acid (red line) bound to HSA; (**C**) detail of SERS spectra shown in the dashed rectangle area shown in (**B**). The chemical structure of the isotopologue used is shown. Spectra of uric acid free and bound to HSA have been obtained from samples of uric acid and HSA (average physiological concentrations) in PBS by centrifugal filtration (cutoff at 3 kDa), recovering the filtrate and the residue, respectively

Although most bands of serum match those of uric acid (Figure 3), an important exception is the band between 1650 and 1660 cm^−1^, not observed in the spectra of any other biomolecule included in this study. In a previous work [1] we discussed this band and tentatively attributed it to a different adsorption geometry of uric acid. Since this band does not appear in a protocol using deproteinized serum [1], we hypothesized that serum proteins (mostly HSA) might have a role in determining uric acid adsorption geometry. Albumins are known to bind uric acid [28–30], so we decided to investigate the effect of HSA on the SERS spectrum of this metabolite. A PBS solution of uric acid and HSA (at the respective average physiological concentrations) was filtered with a 3kDa-cutoff centrifugal filter, and the spectra of both filtrate (i.e., free uric acid) and residue (HSA plus HSA-bound uric acid) were recorded (Figure 4A). Data show that the spectra of these two fractions show some important differences, especially as far as the region between 1650 and 1660 cm^−1^ is concerned. In particular, the protein fraction shows an intense band at 1659 cm^−1^ (similarly to the one observed in the spectra of commercial serum in Figures 1, 2, and 3), whereas the filtrate fraction has a spectrum identical to that of pure uric acid (as expected). The data presented suggest that this band must be due either to protein vibrations or to HSA-bound uric acid. Moreover, conclusive evidence comes from spectra of the same experiment carried out by using an isotopologue of uric acid (i.e., uric acid-2-^13^C, 1,3,7-^15^N_3_), in which three nitrogens and one carbon have been substituted with heavier isotopes. A comparison between the spectra of the protein fraction with unmodified and isotopically substituted uric acid (Figure 4B, C) shows that the band at 1659 cm^−1^ shifts to 1653 cm^−1^, proving that it is due to uric acid, and not to HSA. The relatively small but evident downshift of 6 cm^−1^ is consistent with the assignment of this band to a C=O stretching from the C6 carbon [31] proposed by a previous study. The larger downshifts observed for bands at 867, 1002, 1055, 1125 and 1192 cm^−1^ are also consistent with the previous assignments of these bands to C-N stretching modes.

Thus, data in Fig. 4 show that uric acid can be found in two forms (i.e., free and HSA-bound), having different spectral features, and that the HSA-bound form is responsible for the intense band in the 1650–1660 cm^−1^ region of the SERS spectrum of serum. These differences are likely due to different adsorption geometries for uric acid, since the molecular orientation with respect to the metal surface can affect SERS bands position and relative intensity via both electromagnetic and chemical enhancement mechanisms. A plausible conveyance of the above is that the interaction with HSA is forcing uric acid to adsorb differently on the metal surface. In particular, the higher intensity of the C6=O stretching band for the bound form suggests that this moiety plays a more important role in the interaction with the metal surface, when compared to the free form. Differences in the relative intensities of the bands in the 1100–1400 cm^−1^ region is consistent with a different orientation of C-N bonds of the purine ring system with respect to the surface, while the negligible differences in their Raman shifts suggest the nitrogen atoms are not directly involved in a chemical interaction with the metal.

### SERS spectra of commercial serum spiked with individual biomolecules

A major limitation of the direct spectral comparison to test the spectral contribution of alleged biomolecules to serum is that the matrix effect is neglected. Since SERS intensity depends on the surface-analyte interaction and the formation of hot spots, several aspects linked to serum can play an important role in determining the intensity of a SERS spectrum. For instance, ionic strength, specific ions and the adsorption of other serum components can promote (or hinder, as in the case of proteins [23]) colloidal aggregation and hot spots formation. In addition, some serum components might compete with the analyte for adsorption sites, or they can promote or hinder analyte adsorption via their interaction with the metal or with the analyte itself. All these factors linked to the serum matrix can affect the intensity of a SERS spectrum or even change the overall relative intensity pattern by modulating the adsorption geometry of the analyte. Thus, in principle, the SERS signal of a biomolecule might be detected in serum even if its pure solution does not yield a SERS spectrum. Overall, the relative intensity pattern of a biomolecule in serum might slightly differ from that of the same biomolecule as a pure substance.

**Fig. 5:**
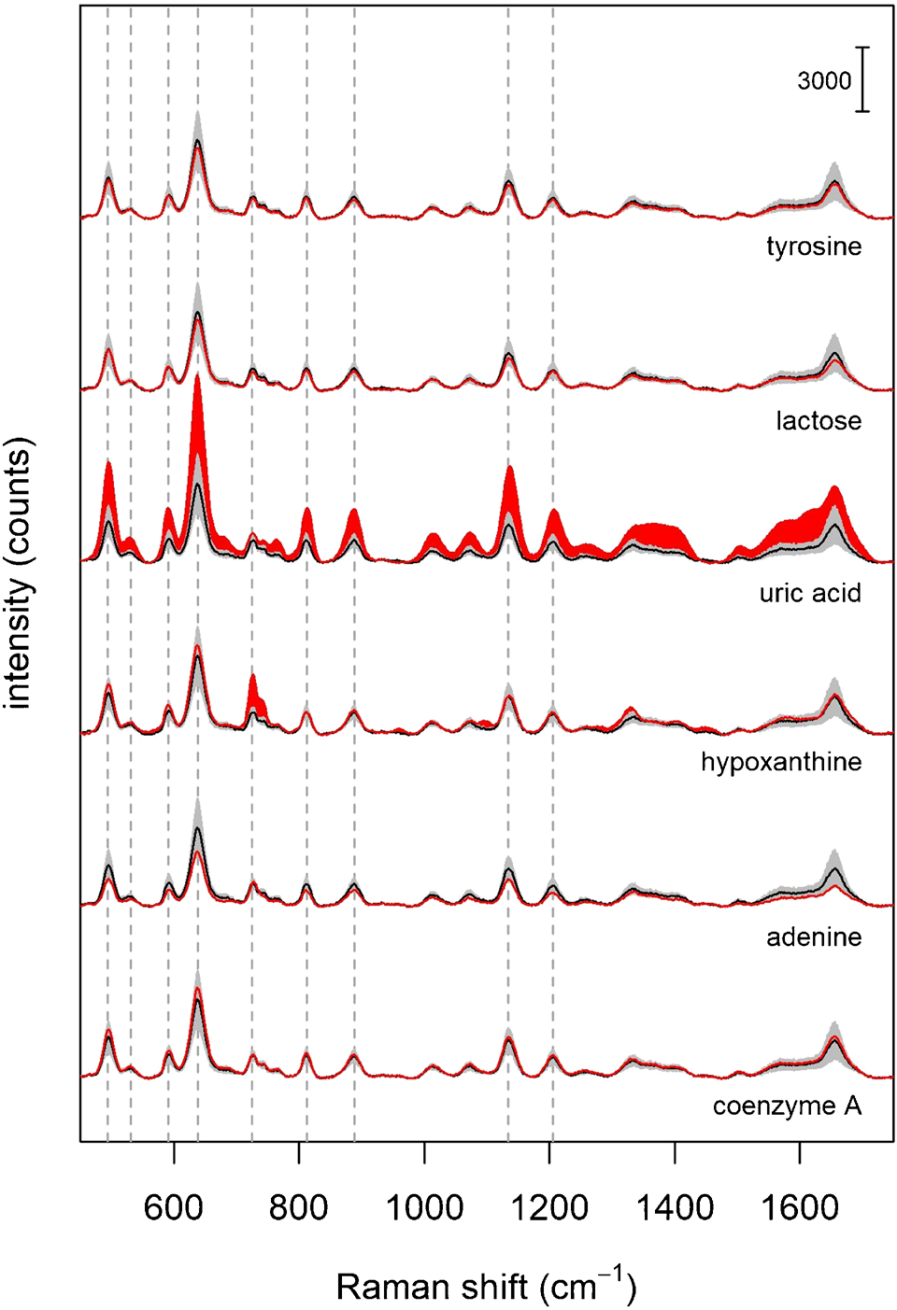
SERS spectra of serum spiked with selected biomolecules (red lines). The concentration increase of the biomolecules in the spiked serum samples are as follows: tyrosine + 55 μM, lactose + 100 μM, uric acid + 280 μM, hypoxanthine + 10 μM, adenine + 0.4 μM, coenzyme A + 100 μM (physiological ranges). The average spectrum of serum is reported for reference (black line), together with the standard deviation of the intensity (gray shaded area). Red areas mark an increase in intensity with respect to the average spectrum of serum (+ 1 s.d.), highlighting the effect of the spike

For this reason, a more reliable method to test the potential contribution of a specific biomolecule to the serum spectrum is to acquire spectra from serum samples spiked with it. This approach has been successfully used by Avci et al. [26] to check the contribution of some amino acids, urea, nucleobases, mannose and xanthine, suggesting that none of these biomolecules is contributing to the serum spectrum. We decided to repeat this experiment, extending it to other biomolecules (Fig. 5 and Figure S9 in Supplementary Material 1).

Results show that, for those biomolecules that are most frequently used for band assignment, spiking does not lead to a significant increase in the SERS intensity of serum bands, with the notable exception of uric acid and hypoxanthine (Fig. 5). The sample spiked with uric acid, in particular, shows an increase of most of the serum bands, further confirming that these bands (including the one at 638 cm^−1^) can be attributed to this metabolite. The sample spiked with hypoxanthine shows an increase for the band at 725 cm^−1^, confirming the attribution initially given by Premasiri et al. [32], based on the direct comparison between spectra of serum and of this metabolite. Notably, the intensity increase of both the 638 cm^−1^ and 725 cm^−1^ bands upon spiking with uric acid and hypoxanthine, respectively, is approximately two-fold, which is compatible with the fact that the final concentrations of metabolites in spiked samples were two-fold higher than the average physiological values. Thus, the data in Fig. 5 (and in Figure S9) seem to confirm that most of the bands of serum are due to uric acid and hypoxanthine, while the other biomolecules, still frequently assigned to serum bands by many authors, are in fact not contributing to the spectrum of serum.

An increase in intensity was also observed for ergothioneine and xanthine (Supplementary Material 1- Figure S9), but at Raman shifts different from those of the average serum bands, meaning that an increase in the serum levels of those biomolecules in real samples is likely to be detected. Ergothioneine bands have been already reported in SERS spectra of serum and other biological samples [3, 33], but apparently it was too low to be detected in the serum samples used for this paper.

Serum samples spiked with HSA (Supplementary Material 1- Figure S9) yield SERS spectra with the same relative intensity pattern of serum, showing that this protein, although having a high concentration in serum (about 600 μM, on average), is not contributing to its spectrum. Moreover, SERS spectra of samples spiked with HSA are significantly weaker, consistently with the hypothesis that serum proteins disfavor metal nanoparticles aggregation and formation of hot spots, likely because of the steric hindrance of protein coronas surrounding nanoparticles [23].

### SERS spectra of commercial serum in presence of uricase

**Fig. 6:**
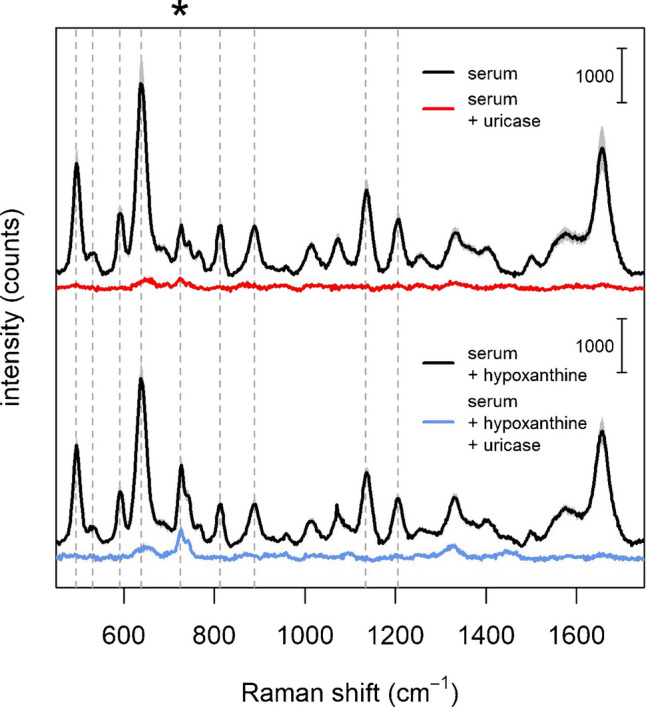
(Upper part) SERS spectra of serum before (black) and after (red) the addition of uricase; (Lower part) SERS spectra of serum spiked with hypoxanthine, before (black) and after (blue) the addition of uricase. Vertical dashed gray lines are corresponding to main serum bands. An asterisk marks the position of the 725 cm^−1^, attributed to hypoxanthine

To further confirm that most bands of the SERS spectrum of serum are due to uric acid, we conducted experiments in which uricase (or urate oxidase), an enzyme oxidizing uric acid to 5-hydroxyisourate, is added to serum [34, 35]. SERS spectra of serum before and after treatment with uricase (Figure 6, top) show that SERS bands are disappearing upon the addition of this enzyme. The suppression of SERS bands, though, might be explained not just with the uric acid depletion, but also by invoking other effects linked to the presence of the enzyme. For instance, being a large protein, the enzyme might interfere with the formation of hot spots, as seen in the case of HSA, thus suppressing the SERS signal altogether. However, SERS spectra of serum spiked with hypoxanthine retain SERS signal due to this metabolite (although reduced in intensity) upon the addition of uricase, while other bands are suppressed (Figure 6, bottom). Therefore, these data indicate that the SERS bands suppressed upon uricase addition are due to uric acid, consistently with results obtained by spiking, and with those from a direct comparison between spectra of uric acid and serum.

### Bands correlation in the donors dataset

**Fig. 7:**
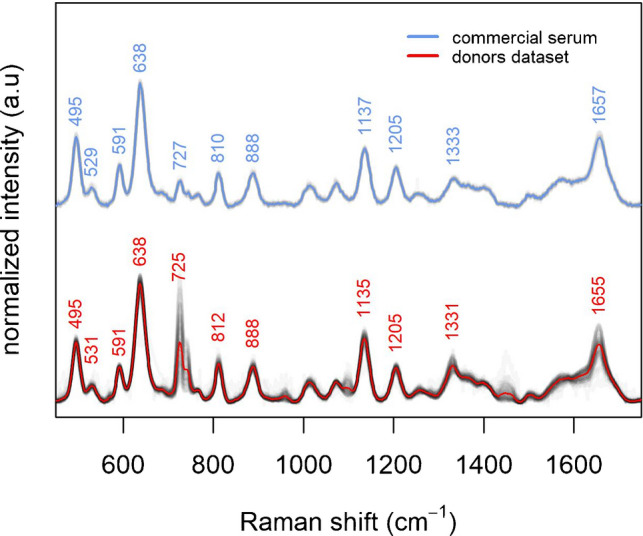
Comparison between SERS spectra (15 replicas) of commercial serum (upper part) and those of serum samples from 81 different donors (lower part). Individual spectra are plotted as transparent gray lines. Average spectra are plotted as colored line (blue for commercial serum, red for donors dataset)

So far, in the present work, SERS data collected from commercial serum from a single donor were presented. Although its spectrum is consistent with those reported in literature by authors using the same protocol, spectral features due to inter-individual variability are missing. To explore inter-individual variability among SERS spectra from different subjects, SERS spectra were collected from serum samples of 81 donors (Figure 7). Spectra from donors samples are also consistent with those from commercial serum, but they present variability in the intensity of some bands, as expected from inter-individual variations in serum metabolic profiles.

**Fig. 8:**
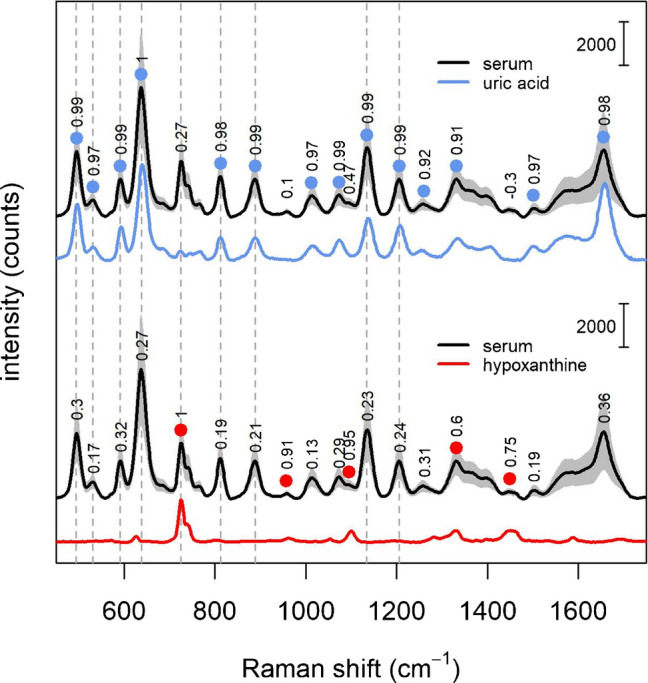
Spearman’s correlation coefficients among the band at 638 cm^−1^ (upper part) or 725 cm^−1^ (lower part) and the other bands in the SERS spectra of serum (black line). Colored dots indicate a moderate-high correlation (> 0.6) with the 638 cm^−1^ (blue dots) or with the 725 cm^−1^ band (red dots). Intensity standard deviation (± 1) for the serum spectra dataset are shown as gray shaded area. Spectra of pure solutions of uric acid (280 μM, blue line) and hypoxanthine (30 μM, red line) are reported for comparison

Investigating the correlation between the intensity of different bands, i.e., determining if the intensity of two bands increase or decrease together in a dataset, as previously done by Buhas et al. [36], is indicative of whether they are due to the same biomolecule or not. Two highly correlated bands, by varying together across different samples, are very likely originating from the same molecular species. The data show that, in the donors dataset, most bands are correlated to the one at 638 cm^−1^ (Fig. 8, upper part). In particular, all bands whose intensity is highly correlated with the 638 cm^−1^ band are coinciding with those of uric acid (reported as reference in Fig. 8, blue line). This indicates that all those bands are due to a single biomolecule, namely uric acid, consistently with the previous results from spiking experiments and uricase experiments, as well as with results reported by Buhas et al. [36]. Conversely, the band at 725 cm^−1^ does not correlate with that at 638 cm^−1^, but it does correlate with other bands (Fig. 8, lower part), which can be observed in the spectrum of hypoxanthine (reported as a reference in Fig. 8, red line). These data are also consistent with those reported by Buhas et al. [36], and with the results of the spiking experiments.

### Fitting serum spectra with uric acid and hypoxanthine

**Fig. 9:**
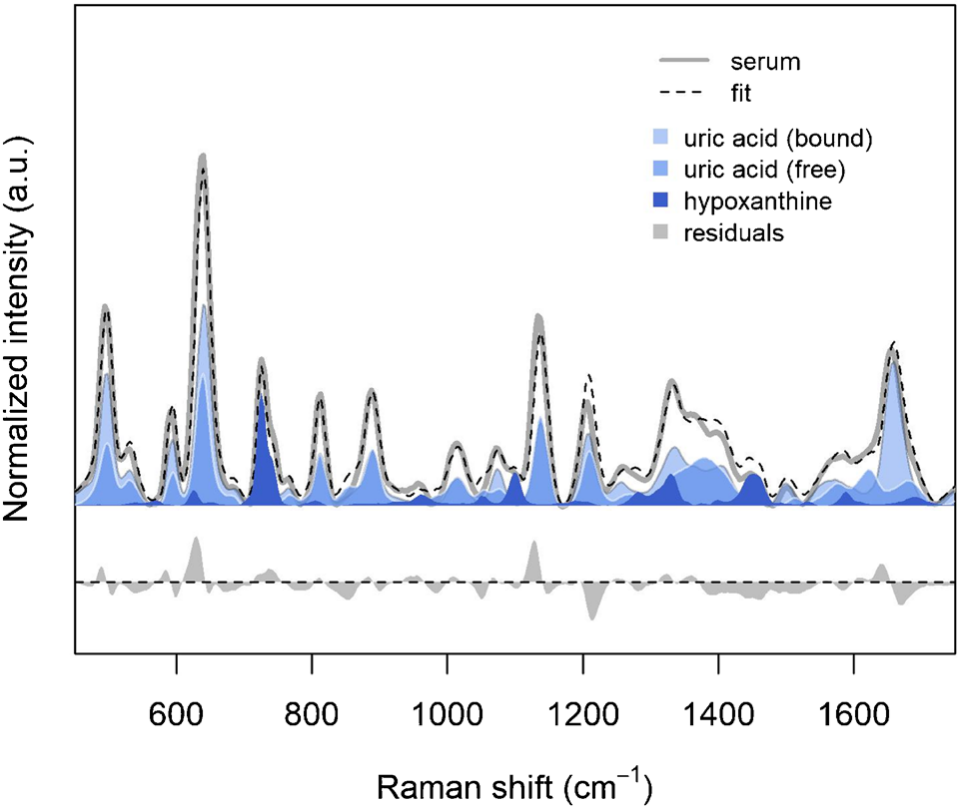
Results of fitting donors serum dataset with spectra of uric acid (free), uric acid (bound to HSA) and hypoxanthine. The gray line represents the median spectrum of the dataset; the dashed black line represents the median of the fitting (as the sum of the three components); the medians of the components are represented as filled areas, each component having a different color. The median of the residuals are shown below (filled gray area). The median figures of merit for the fit, together with their 95% confidence intervals are: R^2^ 0.949 (0.944–0.954), normalized RMSE 0.035 (0.033–0.037), reduced χ^2^ 1.06 (1.05–1.07)

Since the data previously shown point to the fact that uric acid and hypoxanthine are the main contributors to most bands in the SERS spectrum of serum, we further tested this hypothesis by fitting each spectrum of the donors dataset with the spectra of these two metabolites (for uric acid, using both the free and the HSA-bound forms). Serum spectra could be fitted rather well (Figure 9), with an average R^2^ of 0.95, and a reduced χ^2^ of 1, especially when considering the approximations introduced by ignoring any matrix effect on spectra of pure components. Statistics on the fitting coefficients (see Supplementary Material 1 - Figure S10) confirms that uric acid contributes the most to the spectra, the HSA-bound form being the most important. In line with the data previously shown, results from fitting indicate that, even when considering spectra from serum of various donors, SERS spectra of serum can be well explained by invoking the contribution of just two metabolites: uric acid (both free and HSA-bound forms) and hypoxanthine.

### Spectral variability in the donors dataset studied by PCA

**Fig. 10:**
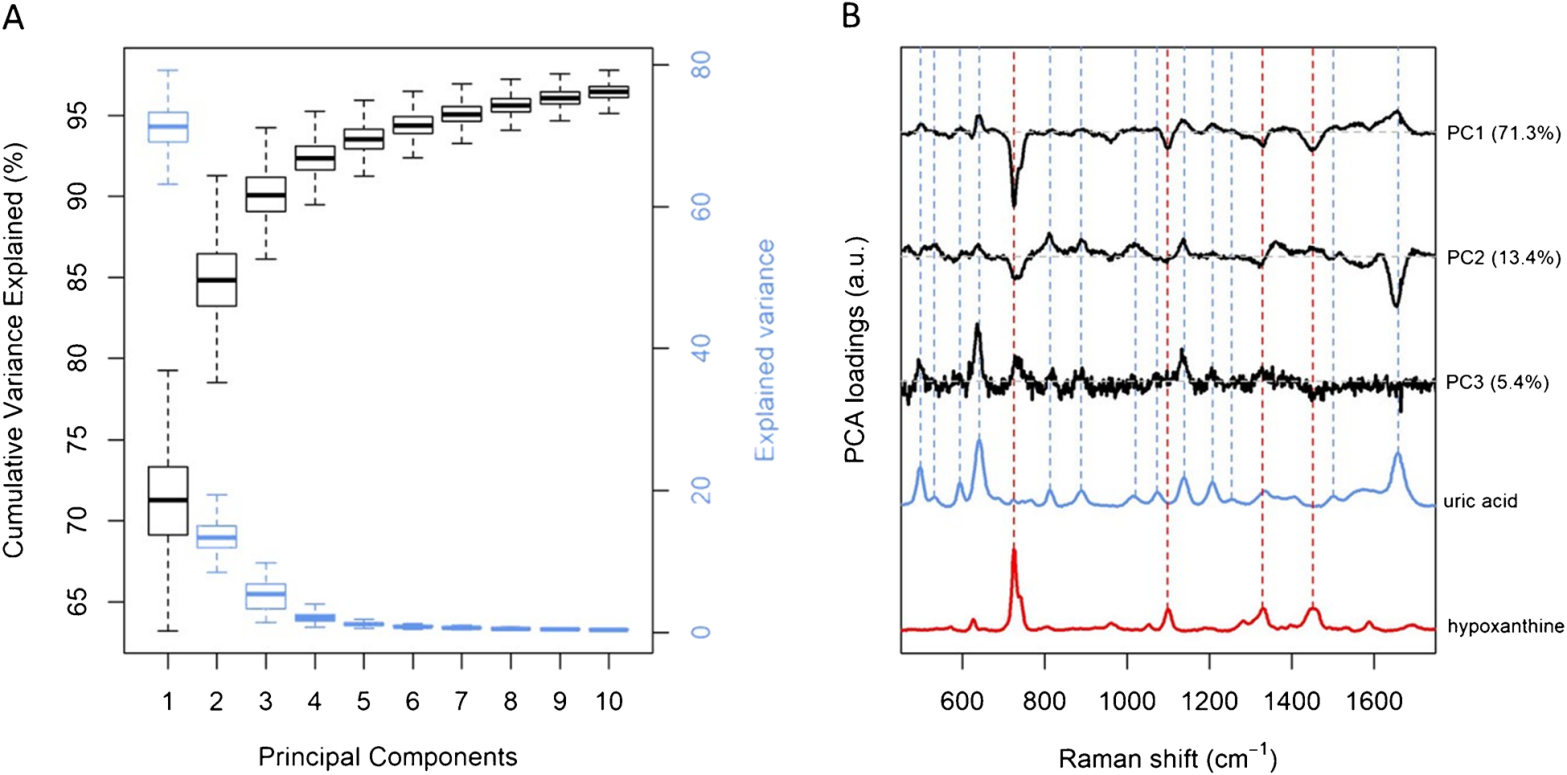
**A** Explained variance (blue) and cumulative explained variance (black) for the first 10 principal components of the donors’ dataset. **B** Loadings (black lines) of the first 3 PCs together with SERS spectra of uric acid (blue line) and hypoxanthine (red line)

Further information from the donors dataset can be inferred by the results of a Principal Component Analysis (PCA). By looking at the variance explained by each Principal Component (PC), together with its loading, one can gain information on how much of the inter-individual variability of SERS spectra of serum is linked to specific bands. PCA results (Figure 10A) show that the first PC explains about 70% of the spectral variability in the dataset, while the first three PCs cover about 90% of the variance. The corresponding loadings (Figure 10B) convey the spectral information about the variance explained by each PC. The largest loadings values for the first three components match the bands of uric acid and hypoxanthine, indicating that 90% of the spectral variability in the dataset is mostly linked to these two metabolites. In particular, the first PC has positive loadings matching the bands of uric acid, and negative loadings matching those of hypoxanthine, showing that more than 70% of the spectral differences in the dataset are due to the relative intensity ratio between the bands of these two metabolites. The second PC has large loadings corresponding to the difference between the two forms of uric acid, suggesting that part of the variability in the dataset can be explained by different relative concentrations of free and HSA-bound uric acid. This is particularly interesting, as the ratio between free and bound uric acid has been linked to some pathologies [30, 37]. The loadings of further PCs (up to 10) are reported in Supplementary Material 1- Figure S11.

## Conclusions

The methods commonly used to attribute SERS bands of serum to specific biomolecules are flawed, for most of the papers considered in the present study. The use of such flawed methods is the main cause of the incongruity found in literature about the biochemical interpretation of SERS spectra of serum, and therefore of the unreliability of any conclusion about biochemical differences among groups of samples. The data reported in the present work by using different experimental approaches prove how the majority of the characteristic bands of SERS spectra of serum (at physiological pH 7.4), obtained on Ag substrates and using a near infrared excitation, are largely due to just two metabolites: uric acid, which contributes to most bands, and hypoxanthine. Data presented showed how the HSA-bound uric acid has a different spectrum than that of its free form, and that both contribute to the SERS spectrum of serum. In particular, the intense band between 1650 and 1660 cm^−1^, frequently reported in literature in SERS spectra of serum, is due to the HSA-bound form of uric acid. Results from both a fitting analysis and a PCA of a dataset of spectra from various donors confirmed that most of the spectral variability, in healthy subjects, is due to uric acid and hypoxanthine. Conversely, the experiments on spiked serum exclude any spectral contribution from most of the biomolecules that are still commonly attributed to serum bands (e.g., tyrosine or lactose, among others). Although contributions from other biomolecules cannot be excluded when studying samples from patients with specific pathologies, the data presented in literature show that most of the serum spectra reported from patients share the same spectral features presented here. These conclusions, moreover, are relevant for blood plasma as well, since its spectrum is essentially identical to that of serum [23], when similar experimental conditions are used. The validity of these conclusions, in fact, is limited to the specific conditions of the protocol used, as it is likely that SERS bands of different molecules might be detected upon using other excitation wavelengths, metal substrates or different pHs. In summary, the data presented in this work settle the dispute on the biochemical interpretation of most of the bands commonly observed in the SERS spectrum of serum, forming the basis for all further studies. The data presented also invite a re-assessment of the previous literature, in particular the biochemical interpretation given to reported spectral differences, based on an incorrect band assignment.

## Supplementary Information

Below is the link to the electronic supplementary material.

Supplementary Material 1 (DOCX 8.73 MB)

Supplementary Material 2 (DOCX 31.5 KB)

Supplementary Material 3 (DOCX 43.1 KB)

Supplementary Material 4 (DOCX 30.1 KB)

Supplementary Material 5 (DOCX 12.7 KB)

## Data Availability

All data generated and analyzed in this study, as well as the R code used to generate all figures, are openly available for download in the Zenodo public repository (zenodo.org) at 10.5281/zenodo.17374939, under a Creative Commons Attribution 4.0 International license.

## Declarations

Informed consent was obtained from all donors to ensure they understood that part of their blood would have been used for research purposes, before blood donation. The present study has been performed in accordance with the ethical standards.
