# Supplementary material for: Adsorption of serum components on Ag colloids: on the biochemical interpretation of surface-enhanced Raman spectra of human serum
Source: Anal Bioanal Chem. 2025 Nov 17;417(30):6823–36. doi: 10.1007/s00216-025-06192-5 (PMC12680727; doi:10.1007/s00216-025-06192-5)
Supplement: Supplementary file 1 — Supplementary Material 1 (DOCX 8.73 MB) [file 216_2025_6192_MOESM1_ESM.docx]

**Supplementary material 1**

**Adsorption of Serum Components on Ag Colloids: On the Biochemical Interpretation of Surface Enhanced Raman Spectra of Human Serum**

Roberto Gobbato^a^, Stefano Fornasaro^b^, Valter Sergo^a^ and Alois Bonifacio^a,^*.

^a^ Raman Spectroscopy Laboratory, Department of Engineering and Architecture, University of Trieste, Via Valerio 6a, 34127, Trieste, TS, Italy

^b^ Department of Chemical and Pharmaceutical Sciences, University of Trieste, Via Licio Giorgieri 1, 34127, Trieste, TS, Italy


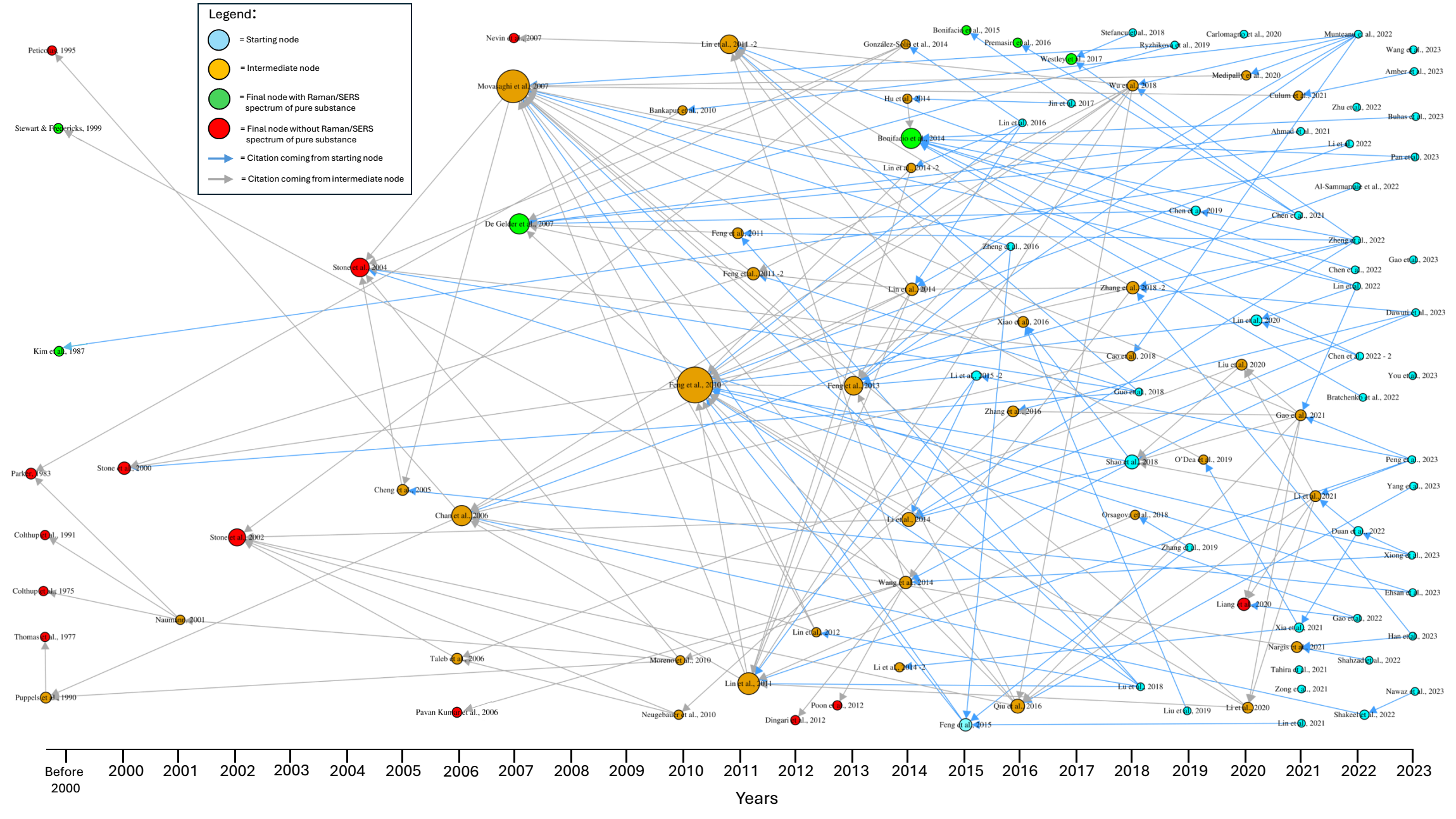


**Figure S1**. Citation network for the 638 cm^-1^ band assignment (see *Materials and Methods* in the paper for a description of the dataset). The correspondent numerical matrix used to build the citation network is provided in **Supplementary material 2 (.xlsx)**.


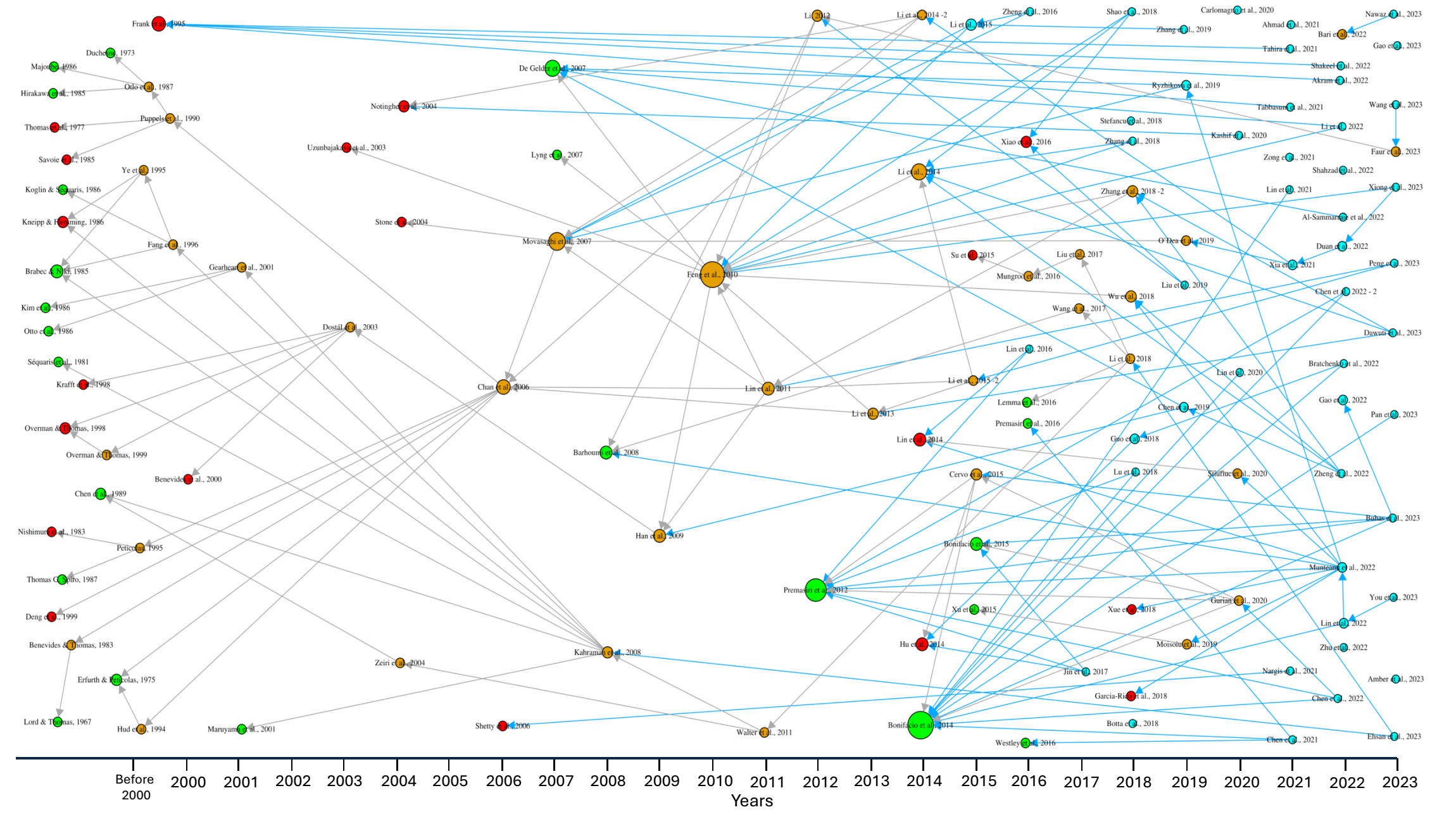


**Figure S2**. Citation network for the 725 cm^-1^ band assignment (see Materials and Methods in the paper for a description of the dataset). The correspondent numerical matrix used to build the citation network is provided in **Supplementary material 3 (.xlsx)**.


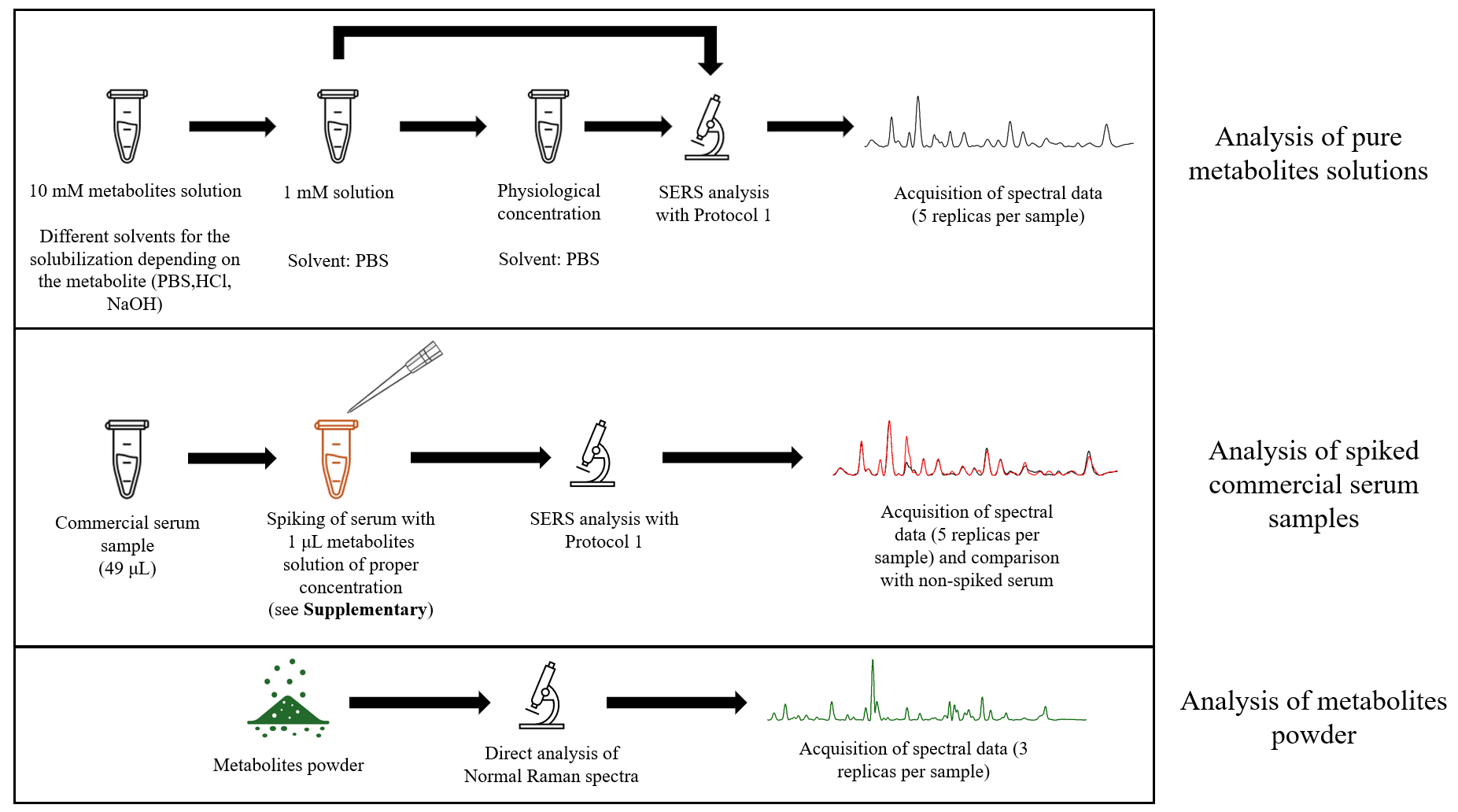


**Figure S3**. Procedures of used for preparation of pure biomolecules solutions at physiological concentrations (top); spiked serum samples (middle); and normal Raman spectra of metabolites powders (bottom). The list of biomolecules analyzed is provided in **Supplementary Material- Table S2 (.docx).**


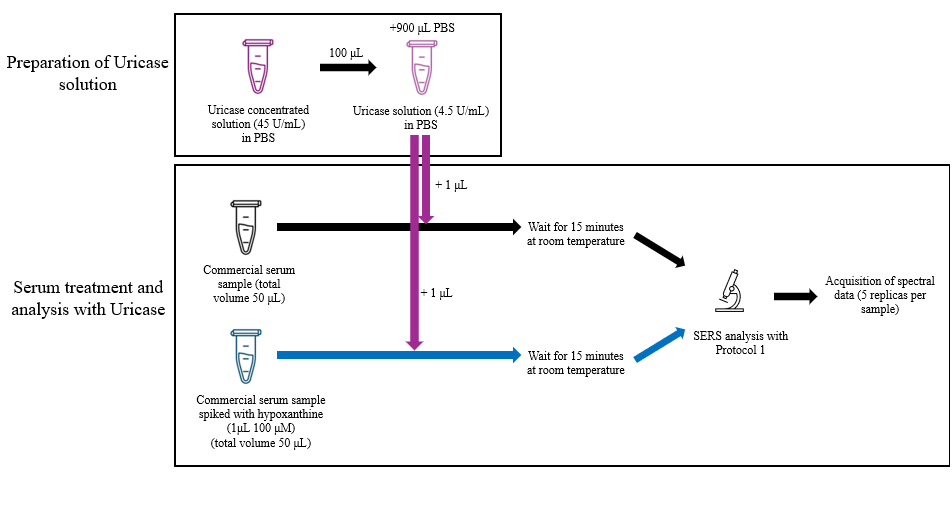


**Figure S4**. Protocol used for uricase experiments.


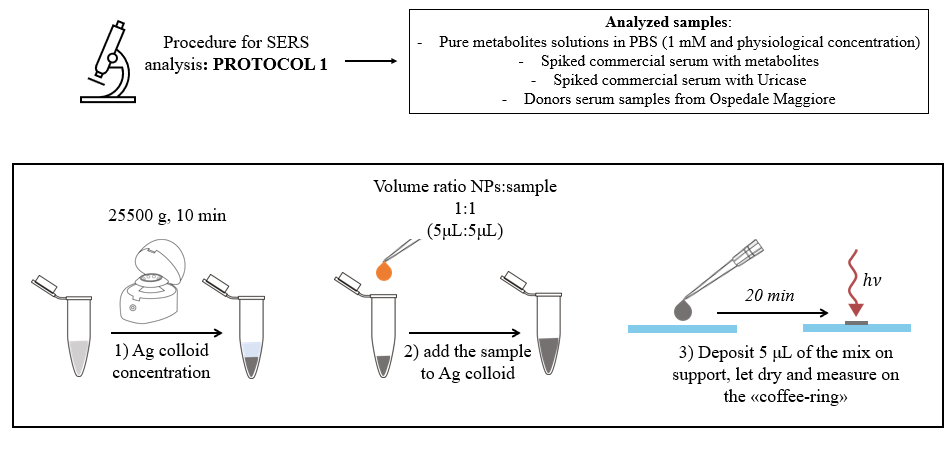


**Figure S5.** Details of the protocol used for SERS measurements.


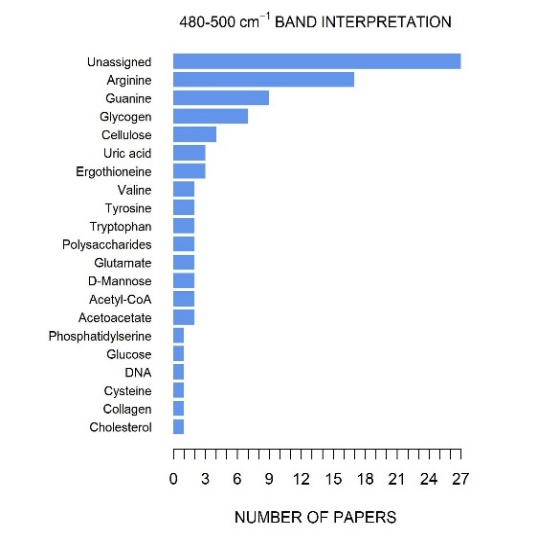

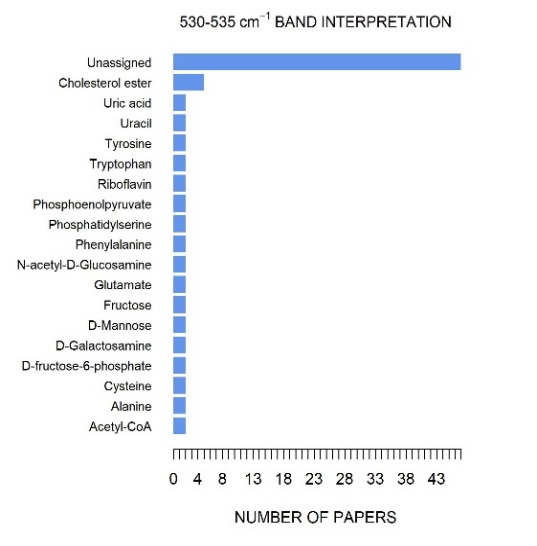

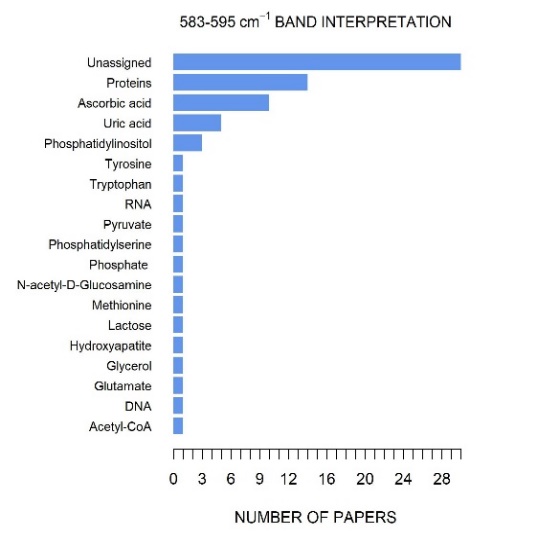

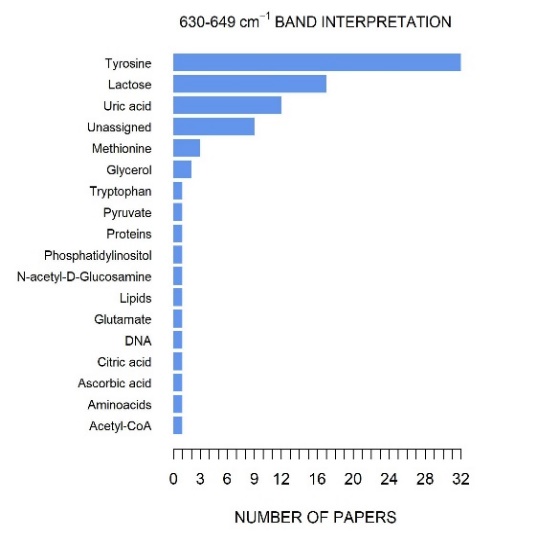

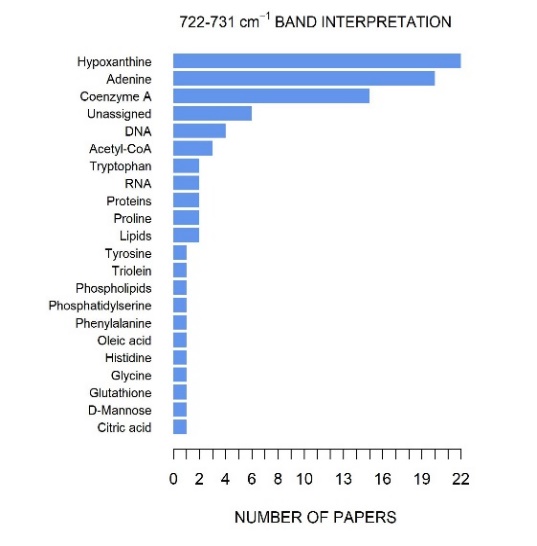

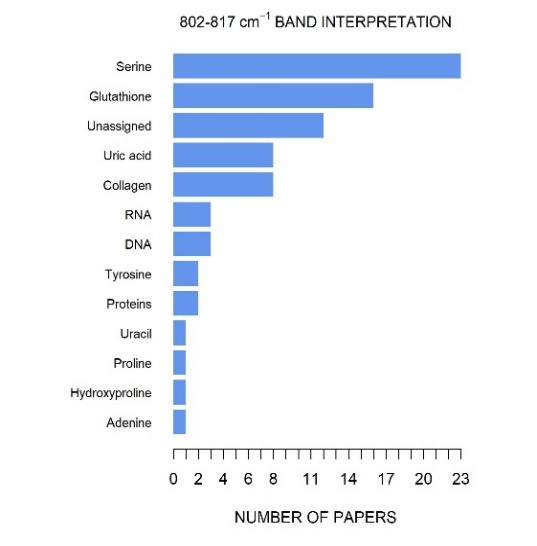

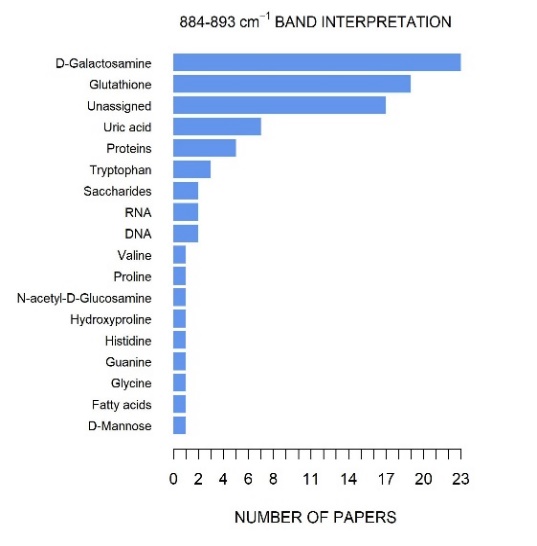

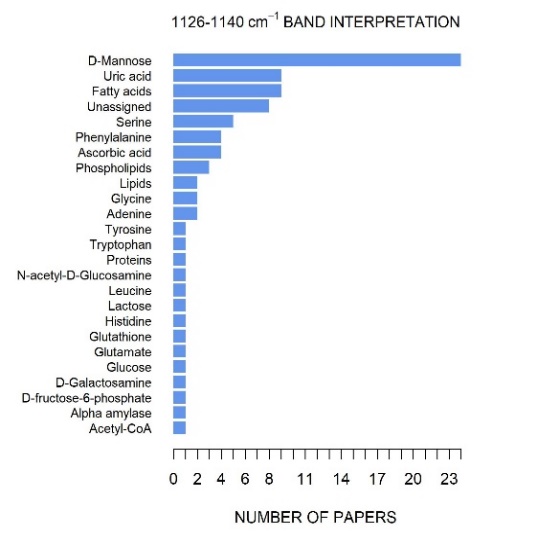

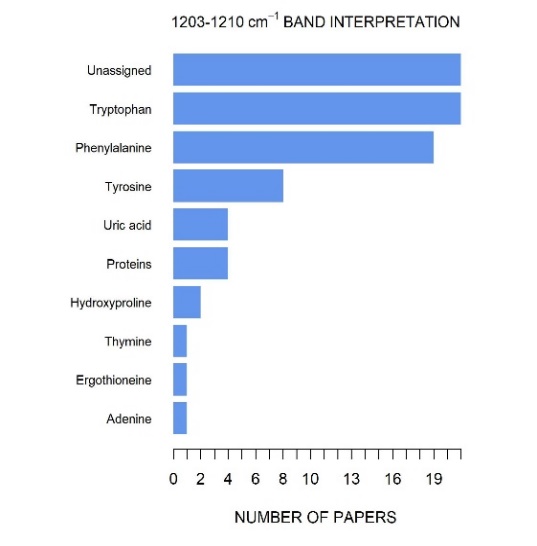


**Figure S6.** Bar plots showing how frequently different biomolecules have been attributed to main bands in the SERS serum of spectrum (i.e. number of papers attributing a specific biomolecule for a specific band). Information of each article are summarized in **Supplementary Material - Table S1**.


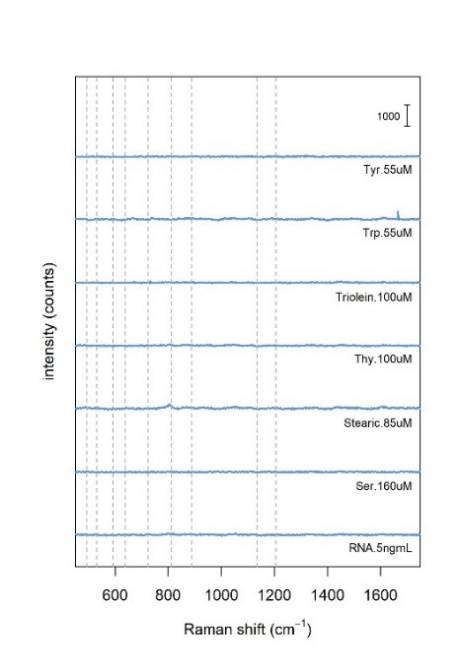

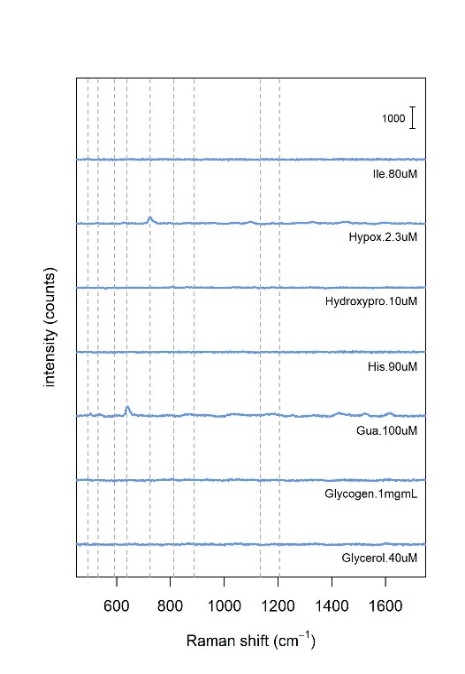

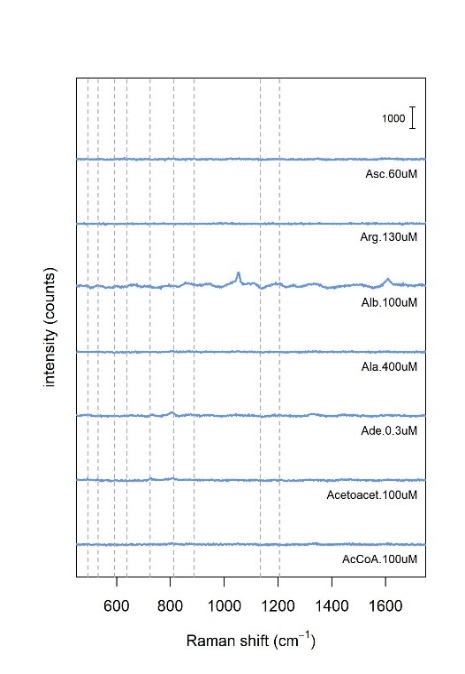

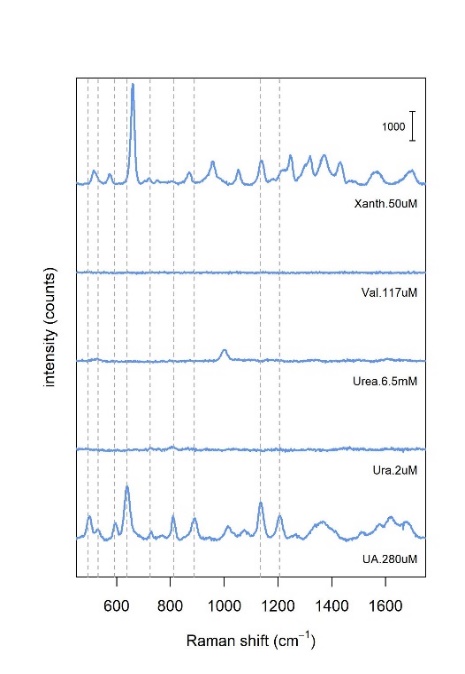

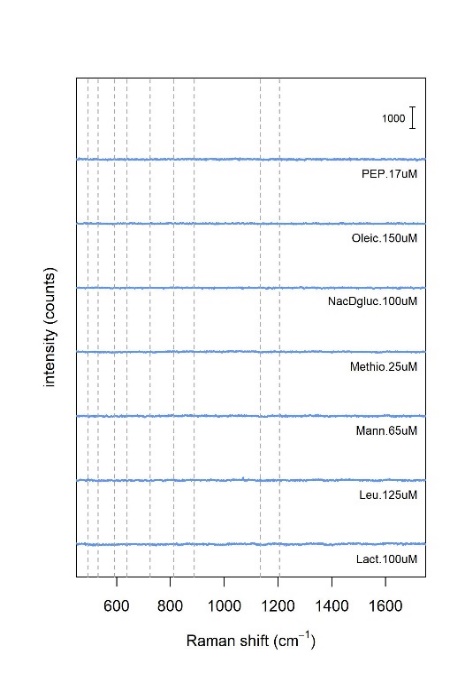

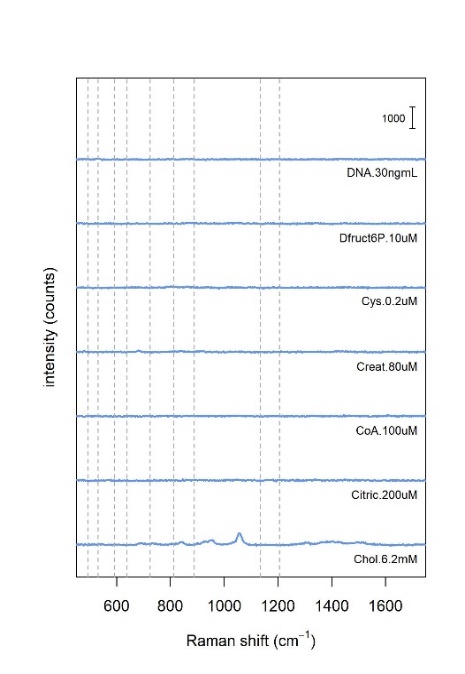

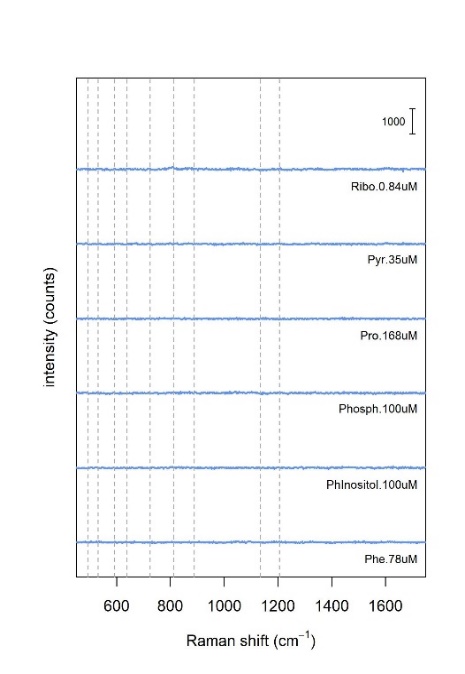

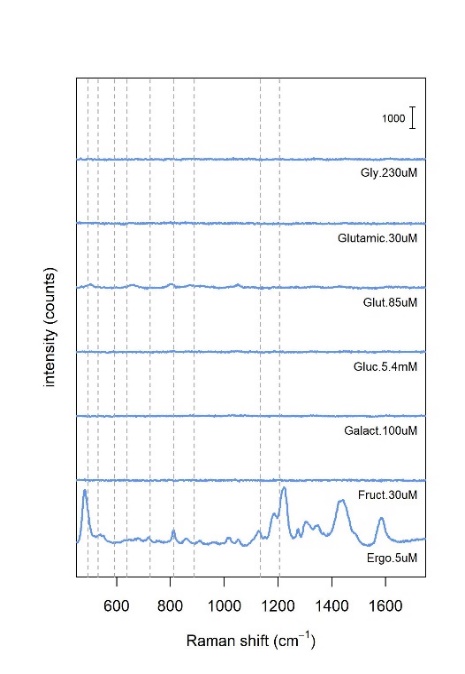


**Figure S7**. SERS spectra of pure biomolecules, at average physiological concentrations in PBS solution.


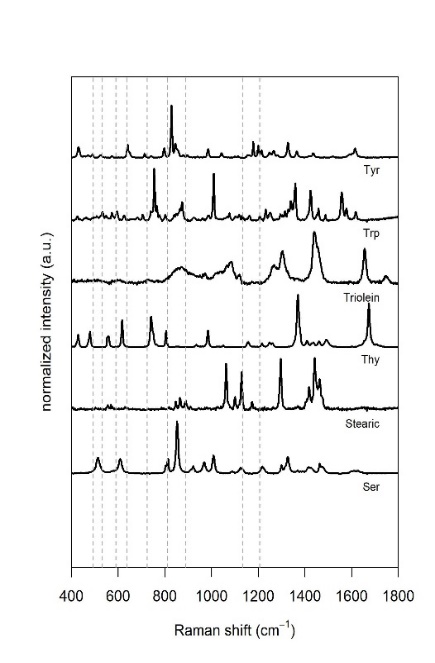

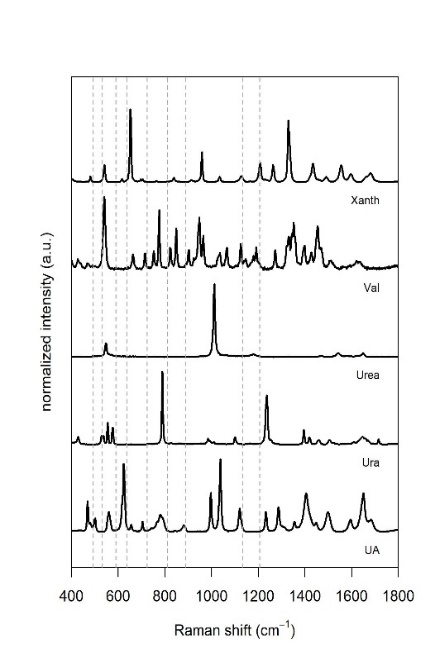

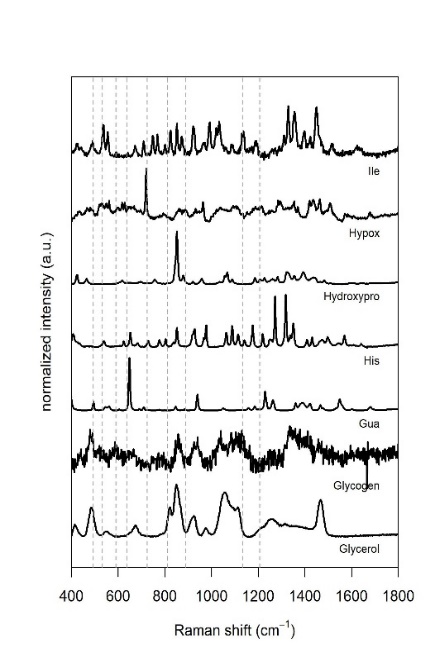

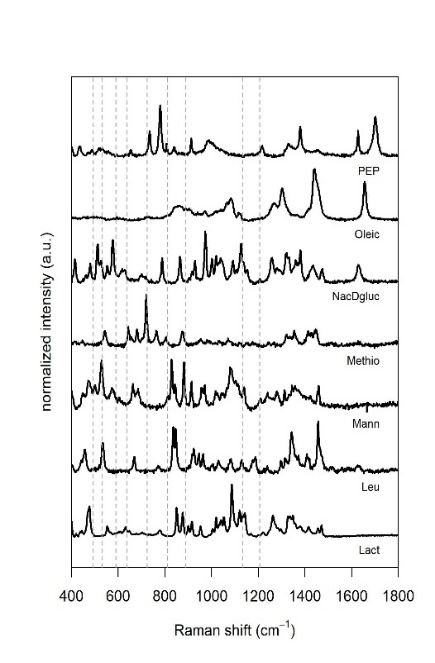

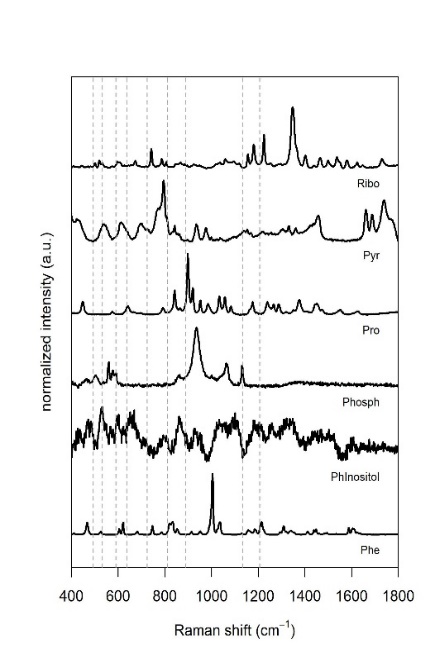

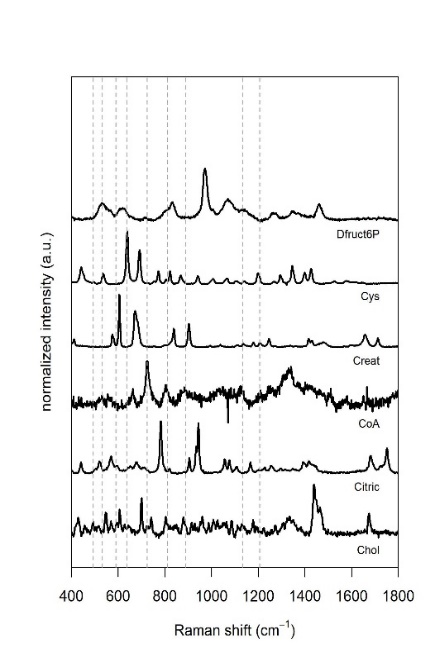

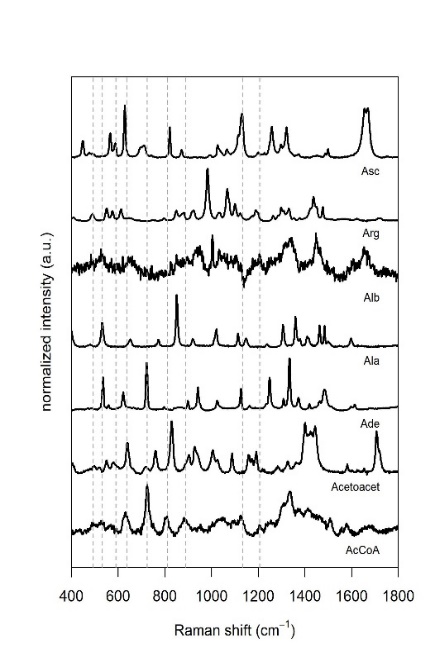

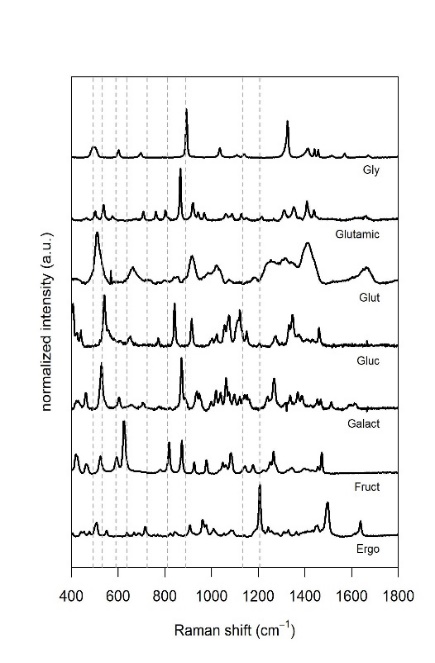


**Figure S8.** Normal Raman spectra of the biomolecules used in **Figures S7** and **S9**.


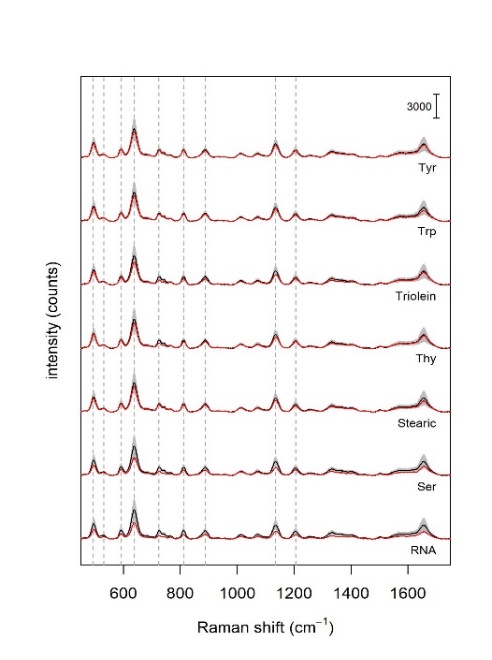

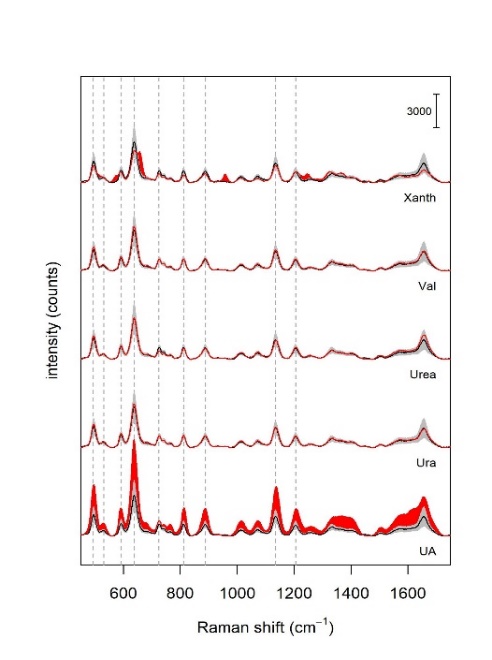

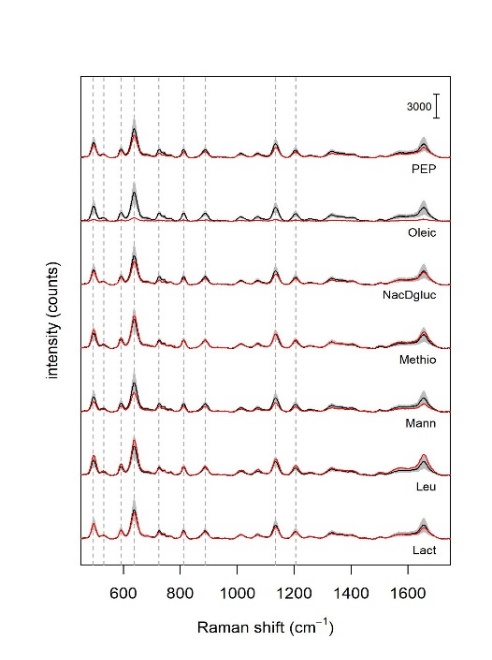

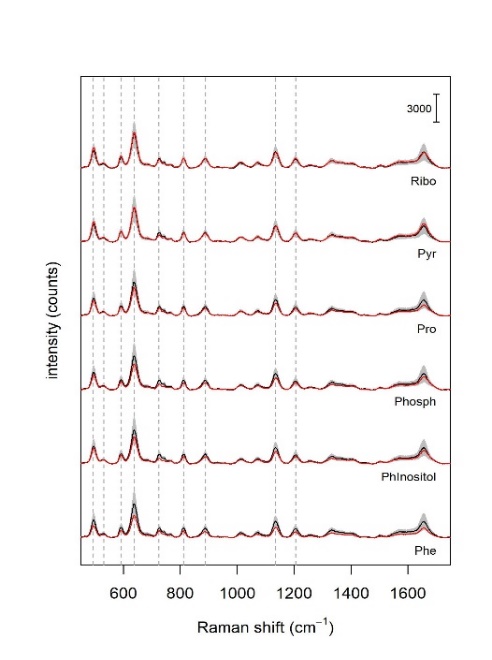

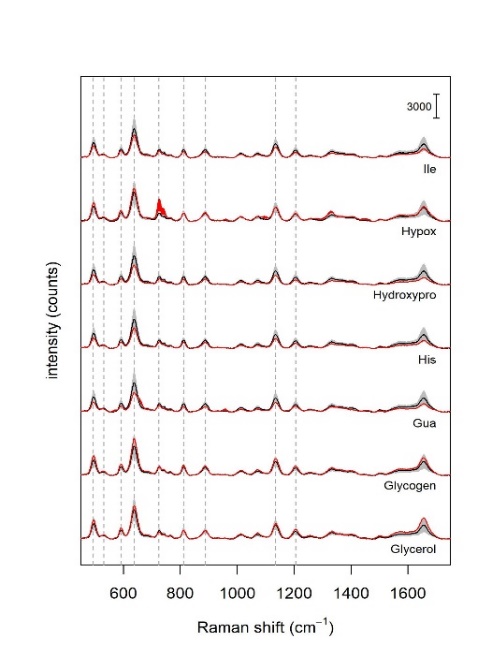

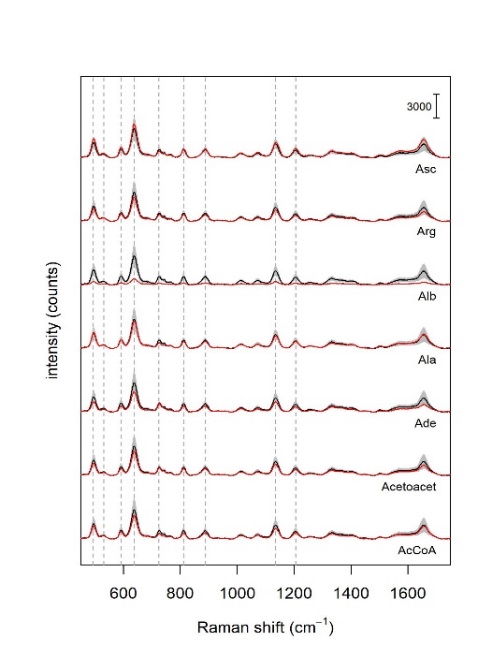

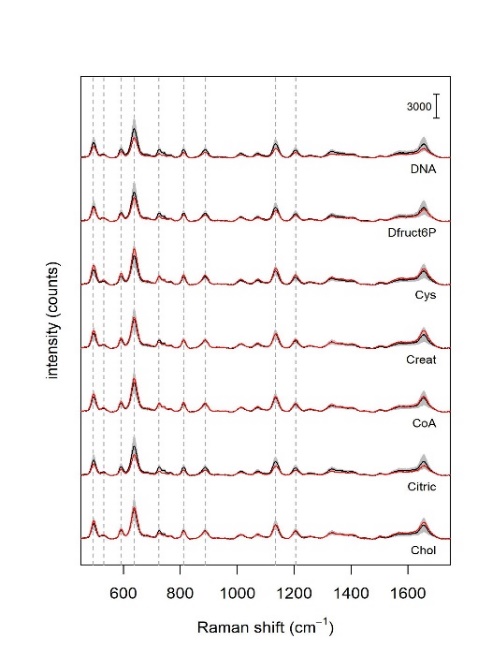

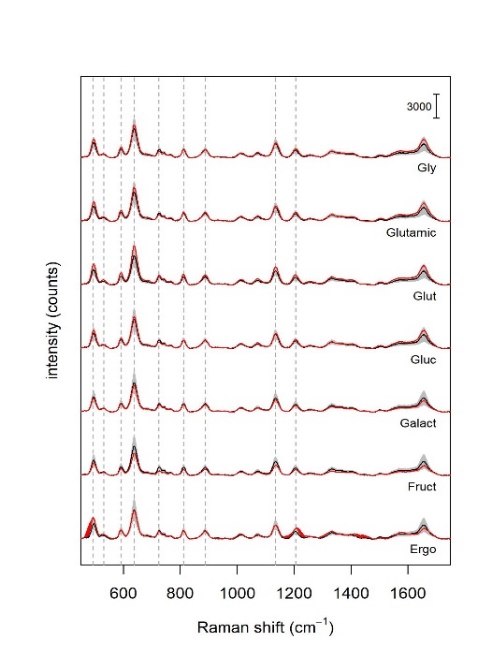


**Figure S9.** SERS spectra of commercial serum spiked with various biomolecules (at a final concentration in serum equal to two times the average physiological concentrations).


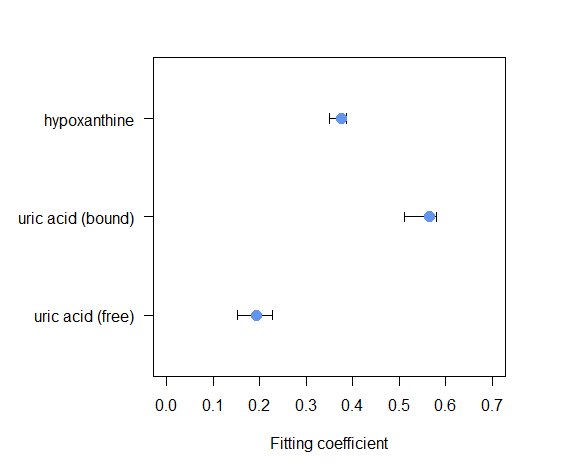


**Figure S10.** Statistics on the fitting coefficients for the components used to fit serum spectra of the 81 donors dataset. Whiskers represent 95% confidence intervals as derived from bootstrapping.


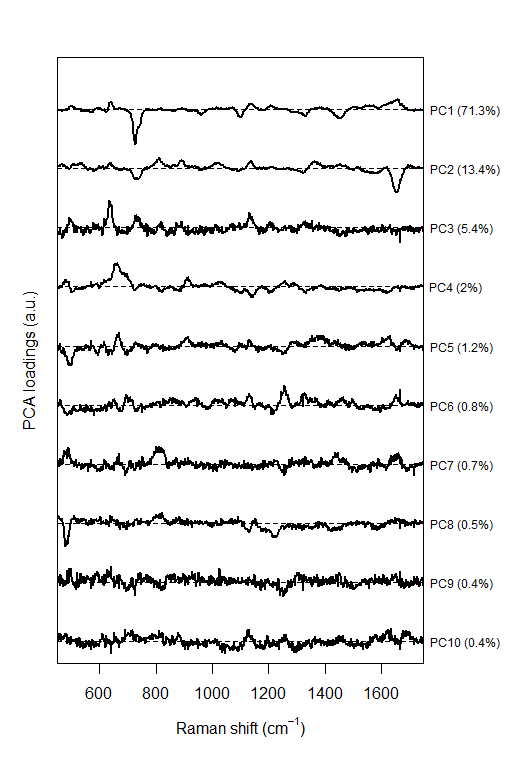


**Figure S11**. Loadings for the first 10 principal components of the serum donors dataset with the correspondent explained variance.
